# Mood and anxiety disorders within the Research Domain Criteria framework of Positive and Negative Valence Systems: a scoping review

**DOI:** 10.3389/fnhum.2023.1184978

**Published:** 2023-06-02

**Authors:** Sarah Jane Böttger, Bernd R. Förstner, Laura Szalek, Kristin Koller-Schlaud, Michael A. Rapp, Mira Tschorn

**Affiliations:** ^1^Social and Preventive Medicine, Department of Sports and Health Sciences, University of Potsdam, Potsdam, Germany; ^2^DZPG (German Center of Mental Health), partner site Berlin/Potsdam, Potsdam, Germany; ^3^Department of Psychiatry, Psychotherapy and Psychosomatics, Brandenburg Medical School, University Hospital Ruppin-Brandenburg, Neuruppin, Germany

**Keywords:** Research Domain Criteria (RDoC), Positive Valence Systems (PVS), Negative Valence Systems (NVS), mood disorders, anxiety disorders, scoping review

## Abstract

**Introduction:**

While a growing body of research is adopting Research Domain Criteria (RDoC)-related methods and constructs, there is still a lack of comprehensive reviews on the state of published research on Positive Valence Systems (PVS) and Negative Valence Systems (NVS) in mood and anxiety disorders consistent with the RDoC framework.

**Methods:**

Five electronic databases were searched to identify peer-reviewed publications covering research on “positive valence” and “negative valence” as well as “valence,” “affect,” and “emotion” for individuals with symptoms of mood and anxiety disorders. Data was extracted with a focus on disorder, domain, (sub-) constructs, units of analysis, key results, and study design. Findings are presented along four sections, distinguishing between primary articles and reviews each for PVS, NVS, and cross-domain PVS and NVS.

**Results:**

A total of 231 abstracts were identified, and 43 met the inclusion criteria for this scoping review. Seventeen publications addressed research on PVS, seventeen on NVS, and nine covered cross-domain research on PVS and NVS. Psychological constructs were typically examined across different units of analysis, with the majority of publications incorporating two or more measures. Molecular, genetic, and physiological aspects were mainly investigated via review articles, primary articles focused on self-report, behavioral, and, to a lesser extent, physiological measures.

**Conclusions:**

This present scoping review shows that mood and anxiety disorders were actively studied using a range of genetic, molecular, neuronal, physiological, behavioral, and self-report measures within the RDoC PVS and NVS. Results highlight the essential role of specific cortical frontal brain structures and of subcortical limbic structures in impaired emotional processing in mood and anxiety disorders. Findings also indicate overall limited research on NVS in bipolar disorders and PVS in anxiety disorders, a majority of self-report studies, and predominantly observational studies. Future research is needed to develop more RDoC-consistent advancements and intervention studies targeting neuroscience-driven PVS and NVS constructs.

## 1. Introduction

Mood and anxiety disorders are highly prevalent and comorbid (Jacobi et al., 2014; World Health Organization, 2017) with mood disorders including major depression (MDD), dysthymia, bipolar disorder I and II (BD). Anxiety disorders (AD) comprise panic disorder (PD), agoraphobia (AG), generalized anxiety disorder (GAD), social anxiety/phobia (SAD), and specific phobia (SPD) and together with depressive disorders are amongst the major contributors of global disease burden (World Health Organization, 2017), with mood and anxiety disorders affecting ~8.3% of the total global population in 2019 (Global Health Data Exchange, 2020). Regarding the various diagnostic categories within mood and anxiety disorders, research has reported a substantial overlap in phenomenology and neurobiological mechanisms (Kendler et al., 1992; Watson, 2005). Especially for these disorders, there are challenges to the neurobiological phenotypic and diagnostic specificity that would be essential to refine treatments to ultimately improve treatment response in mental illness (Insel et al., 2010).

The United States (US) National Institute of Mental Health (NIMH) initiated the Research Domain Criteria (RDoC) project in 2010 to address the above mentioned issue of limited specificity and to offer a new framework to investigate mental disorders. The RDoC initiative had been developed to guide research on mental disorders with reference to disrupted brain and behavioral mechanisms, in contrast to “classifying non-taxonic [sic], multidimensional phenomena […] as mental disorders” (NIMH, 2008; Cuthbert and Insel, 2010, 2013; Clark et al., 2017, p. 94). Providing a dynamic guiding framework for research, the idea of the dimensional approach of RDoC has been to understand mental illness in all its complexity, therefore studying the full range of human functioning from normal to abnormal with respect to basic circuit-based behavioral dimensions, organized into major systems of emotion, cognition, motivation, and social behavior (Cuthbert and Insel, 2013; Clark et al., 2017; NIMH, 2023a). The NIMH's hope is that the RDoC framework will help to generate research that enables an improved characterization within this multidimensionality (Clark et al., 2017). The RDoC framework is conceptualized as a matrix currently grouped into six basic domains of functioning: Positive Valence Systems (PVS), Negative Valence Systems (NVS), Cognitive Systems (CS), Social Processes (SP), Arousal and Regulatory Systems (ARS), and Sensorimotor Systems (SmS; Insel et al., 2010; NIMH, 2023a,b). These domains can be investigated using the following units of analysis: Genes, molecules, cells, circuits, physiology, behavior, and self-report. The six domains are divided into constructs of which some are again divided into subconstructs. Within the RDoC framework there is great flexibility regarding the use of measures within each domain and regarding the units of analysis to allow for the investigation of all constructs that are relevant to improve knowledge about the etiology of mental diseases (Cuthbert, 2014; Clark et al., 2017).

The two domains of PVS and NVS and their corresponding constructs and subconstructs are particularly relevant to mood and anxiety disorders, as these systems are also represented in the Tripartite Model of Anxiety and Depression (Clark and Watson, 1991). Specifically, the model posits that NVS is predominant in anxiety disorders, and for PVS, alterations in hedonia may be more specific to mood disorders, while depressed mood has been shown to be present in both mood and anxiety disorders. The PVS domain encompasses systems that are “responsible for responses to positive motivational situations or contexts” (NIMH, 2023b). The PVS domain is currently grouped into the constructs reward responsiveness, reward learning, and reward valuation. Subconstructs within these constructs are reward anticipation, initial response to reward and reward satiation for reward responsiveness, probabilistic and reinforcement learning, reward prediction error and habit for reward learning, reward probability, delay and effort for reward valuation. The NVS domain covers systems that are “primarily responsible for responses to aversive situations or contexts” (NIMH, 2023b). The NVS domain currently encompasses the constructs acute threat (fear), potential threat (anxiety), sustained threat, loss, and frustrative nonreward. These constructs are not further divided into subconstructs.

While there has been a growing body of research adopting RDoC-related methods and constructs since its launch in 2010, there is a lack of comprehensive reviews providing an overview of published empirical research consistent with the RDoC framework (Carcone and Ruocco, 2017) and its dimensional and transnosological view on specific symptoms prevalent in existing diagnostic categories. Therefore, by changing the perspective from a focus on disease categories to broader RDoC domains, our goal was to bring together the existing research from this period into one review, which specifically focuses on overlapping constructs that are associated with comorbid and overlapping symptoms. Specifically, the purpose of this study was to conduct a scoping literature review to systematically summarize research investigating PVS and NVS constructs of mood and anxiety disorder symptoms as an approach toward the RDoC system. The following research question was formulated: What is the state of published research investigating the role of PVS and NVS with respect to mood and anxiety disorder symptoms using the RDoC framework? We hypothesized that this scoping review would add insight into the heterogenic diagnostic category of mood and anxiety disorders from the RDoC perspective and therefore enrich our basic understanding of the similarities and differences within this disease spectrum.

## 2. Methods

### 2.1. Review approach

The present scoping review was conducted in accordance with the Joanna Briggs Institute (JBI) specific recommendations for conducting scoping reviews (Arksey and O'Malley, 2005) and the Preferred Reporting Items for Systematic Reviews and Meta-Analyses extension for Scoping Reviews (PRISMA-ScR) guidelines (Tricco et al., 2018; see checklist in Supplementary material A). The objectives, inclusion criteria, and methods for this scoping review had been specified in advance and had been documented in our protocol (see Supplementary material B).

### 2.2. Eligibility criteria

Articles were included if the following inclusion criteria were met: (1) research with outcome measures of positive or negative valence with reference to the RDoC PVS and NVS framework, (2) research on all RDoC units of analysis, which are genetic, molecular, cellular, circuitry, physiological, behavioral and self-report assessments, (3) human studies of adult (18 years and older) participants, (4) individuals with symptoms of mood (depression, bipolar) or anxiety (anxiety or phobic) disorders, (5) all types of empirical research, (6) published in peer-reviewed journal papers, and (7) with full texts available. There were no language restrictions.

### 2.3. Information sources and search

We systematically searched the five electronic databases PubMed, PsychInfo, PsychArticles, PSYNDEX, and Web of Science first on April 26, 2021 and again on January 21, 2023. The search was conducted at domain level of PVS (keywords “positive valence”) and NVS (“negative valence”) and using the search terms “valence,” “affect,” and “emotion.” Our intention was to provide a more comprehensive coverage of search results, as authors in the initial RDoC publications referred to “positive affect,” “negative affect,” “positive emotionality” (Sanislow et al., 2010, p. 634) or “negative emotionality” (Insel et al., 2010, p. 749) when discussing potential areas of research that might have links to psychopathological mechanisms. The final search strategy for PsychInfo is presented in Table 1. For detailed search strategies for all sources, see Supplementary material C.

**Table 1:** PsychInfo search strategy.

**Search component**	**Search terms^a^**
**Search 1**	
S1	AB (“depression” or “depressive disorder^*^” or “depressive symptom^*^” or “major depressive disorder”) OR AB “affective disorder^*^” OR AB “mood disorder^*^” OR AB (“bipolar disorder^*^” or “bipolar” i or “bipolar ii” or “manic depression” or “bipolar affective disorder^*^” or “bipolar depression”) OR AB (“mania” or “manic” or “manic episode”) OR AB (“anxiety disorder^*^” or “anxiety”) OR AB (“phobia” or “phobic disorder^*^”) OR AB (“panic disorder^*^”)
S2	AB “rdoc” OR AB “research domain criteria”
S3	AB “positive valence” OR AB “negative valence”
S4	S1 AND S2 AND S3
**Search 2**	
S5	AB “valence” OR AB “affect^*^” OR AB “emotion^*^”
S6	S2 AND S3 AND S5
**Conjunction of Search 1 and Search 2**	
S7	S4 OR S6

AB, Abstract; rdoc, Research Domain Criteria.

a The asterisk symbol (*) serves as a placeholder, enabling the inclusion of word variations and multiple endings in the search results.

### 2.4. Selection of sources of evidence

To identify relevant articles, a total of four members of our research team rated the articles independently, with two raters at each screening stage. We exported the search results into Citavi (version 6.14.4) and Covidence software. Both software programs detected and removed duplicates. Citavi was used to organize the extracted publications, while Covidence was used for the management of the search results, study selection, and data extraction. The study selection was carried out in two stages. First, we screened titles and abstracts of all articles against the eligibility criteria. Screening of titles and abstracts was performed with Covidence, alongside with double-checking references in Citavi and Microsoft Excel (version Microsoft 365) to ensure high quality of our review. In a second step, we examined full texts for all articles that were potentially relevant to our research objective. Disagreements between raters at each step were resolved by consensus after reviewing the full text.

### 2.5. Data charting and synthesis of results

If an article was eligible for inclusion in this study, we extracted data with focus on disorder, domain and constructs assessed, units of analysis, main aim, key findings, and general information including first author, year of appearance, origin (country/language), and study design. In line with scoping review guidelines, risk of bias assessment was not carried out (Tricco et al., 2018). The included studies were heterogeneous in terms of the outlined extracted information. We grouped sources by type of domain and study design and mapped information from the articles to the type of disorder, the seven units of analysis, and RDoC constructs. In addition, we listed relevant empirical elements and reported the key findings of the publication (see **Tables 3**–**8**). As a relevant number of articles was cross-domain oriented and this approach may shed light on differential effects of PVS and NVS on mood and anxiety disorder symptoms, we grouped those findings in a cross-domain section.

## 3. Results

### 3.1. Selection of sources of evidence

**Figure 1:**
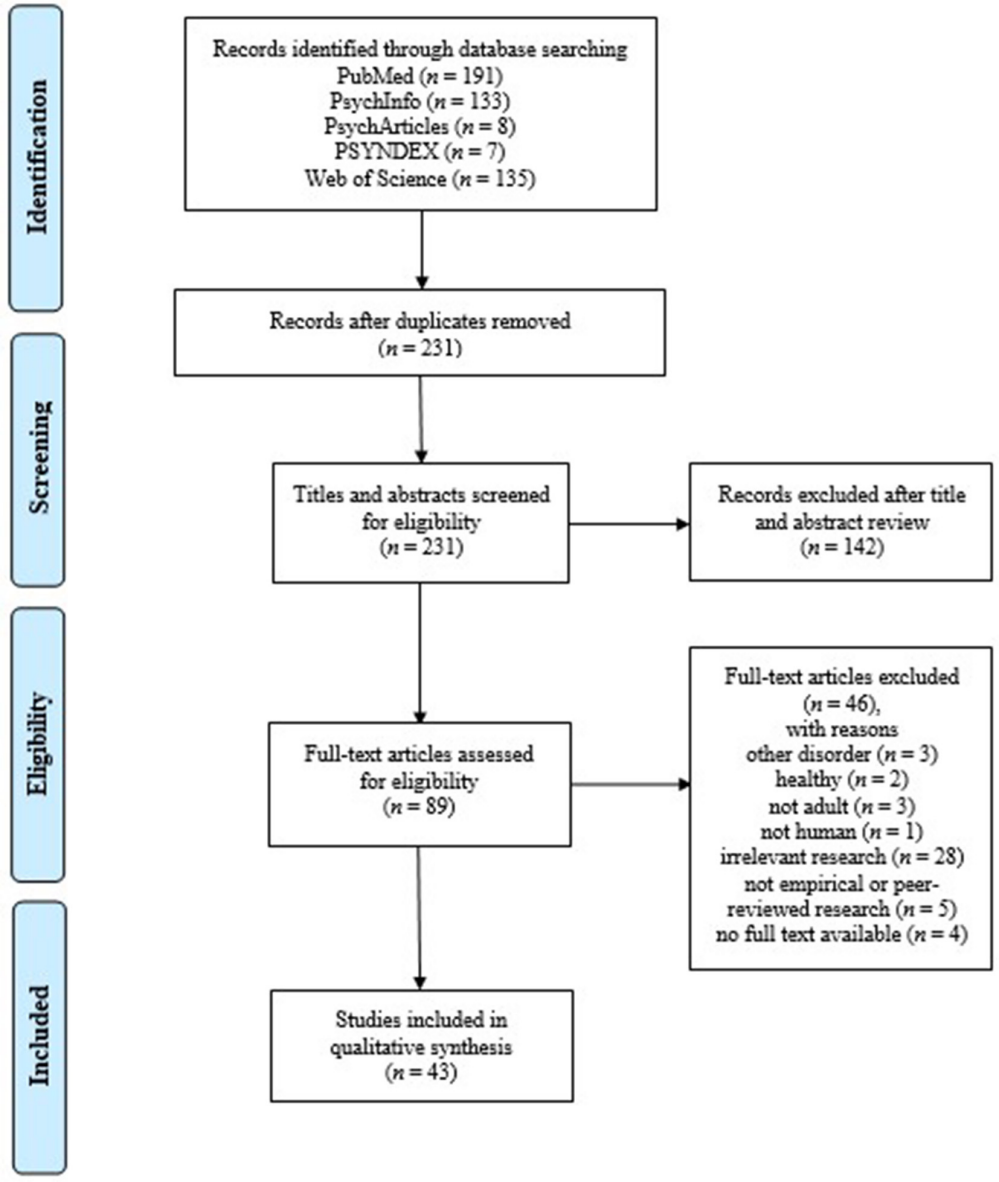
PRISMA flow diagram.

After duplicates were removed, we identified a total of 231 citations from searches of the five electronic databases. Based on title and abstract, 142 publications were excluded, with 89 full text papers to be assessed for eligibility. Of these, 46 were excluded. The remaining 43 studies were considered eligible for this scoping literature review (for reference lists of all papers searched, see Supplementary material D). The flow diagram is shown in Figure 1.

### 3.2. Characteristics of sources of evidence

The characteristics of the 43 included studies are presented in Table 2. The majority of sources were conducted in the US (*n* = 33, 75 %), three studies in Germany, two in Canada, and one study each in Australia, Brazil, Norway, the Netherlands, and the United Kingdom (UK). All articles were written in English. In this final scoping review, we identified 23 primary studies and 20 reviews addressing PVS and NVS in patients suffering from mood and anxiety disorders published between 2014 and 2023. Most of the papers included in this review reported results from self-report questionnaires and interviews (59%; 22 primary articles, four reviews), half used physiological measures (50%; eight primary articles, 14 reviews), and 19 included behavioral indicators (43%; 10 primary articles, nine reviews). Circuitry played a role in about a third of the reviewed publications (32%; five primary articles, 12 reviews). Not as strongly represented were cellular (7%; three reviews), molecular (11%; one primary article, four reviews), and genetic (11%; one primary article, four reviews) components. Findings are presented separately for PVS, NVS, and cross-domain studies (Tables 3–8).

**Table 2:** Characteristics of sources included.

**References**	**Origin^a^**	**Design**	**Disorder^b^**	**RDoC domain(s)**	**RDoC construct(s)**	**RDoC units of analysis**
**Alexopoulos et al. (2015)**	**US**	**Proof of concept study**	**MDD**	**PVS**	**Reward learning Reward valuation**	**Behavior Self-report**
Alexopoulos et al. (2016)	US	Cohort study	MDD	PVS	Reward learning Reward valuation	Behavior Self-report
Barch et al. (2016)	US	Literature review	MDD	PVS	Initial responsiveness to reward Reward anticipation or expectancy Incentive or reinforcement learning Effort valuation Action selection	Circuits Behavior
Baskin-Sommers and Foti (2015)	US	Literature review	MDD	PVS	Reward processing: Initial responsiveness to reward Reward valuation (approach motivation) Reward learning (habit)	Cells Circuits Physiology
Boecker and Pauli (2019)	Germany	Literature review	MDD, AD	NVS	Threat processing: Acute (fear), Potential (anxiety) Sustained threat	Physiology
Cochran et al. (2020)	US	Cohort study	MDD, AD	NVS	Potential threat (anxiety) Loss Frustrative nonreward	Self-report
Ellingson et al. (2016)	Australia	Cohort study	MDD	NVS	Potential threat (anxiety)	Genes Self-report
Ethridge et al. (2021)	Canada	Case control study	MDD	PVS	PVS functioning	Physiology Self-report
Fettes et al. (2017)	Canada	Literature review	MDD	PVS	Reward learning Reward valuation (reappraisal)	Circuits Physiology
Förstner et al. (2022)	Germany	Cohort study	MDD, BD, AD	PVS, NVS	Reward responsiveness Reward learning Potential threat (anxiety)	Behavior Self-report
Gibb et al. (2016)	US	Literature review	MDD, AD	NVS	Sustained threat Loss	Genes Circuits Physiology Behavior
Gruber et al. (2015)	US	Case control study	MDD, BD	PVS	Positive affectivity Reward responsiveness (initial response to reward)	Physiology Self-report
Guineau et al. (2022)	Netherlands	Cohort study	MDD, AD	NVS	Loss (anhedonia)	Self-report
Gunzler et al. (2020)	US	Cohort study	MDD	NVS	Loss (anhedonia, guilt, and self-harm)	Self-report
Hamm et al. (2016)	Germany	Literature review	AD	NVS	Threat processing: Acute (fear) Potential (anxiety) Sustained threat	Genes Physiology Behavior
Janiri et al. (2020)	US	Meta-analysis	MDD, BD, AD	PVS, NVS	Reward responsiveness Reward valuation Acute threat (fear) Potential threat (anxiety) Frustrative nonreward	Circuits Physiology
Khazanov et al. (2020)	US	Validation study	MDD	PVS	Reward processing (anticipation, responsiveness, learning, valuation, satiation, and anhedonia)	Self-report
Klumpp and Shankman (2018)	US	Literature review	MDD, AD	NVS	Threat processing: Acute (fear) Potential (anxiety) Sustained threat	Physiology
Lang et al. (2016)	US	Cohort study	MDD, AD	NVS	Acute threat (fear)	Circuits Physiology Behavior Self-report
Lang et al. (2018)	US	Cohort study	MDD, AD	NVS	Acute threat (fear)	Physiology Self-report
Langenecker et al. (2014)	US	Literature review	MDD, BD	PVS, NVS	Reward and loss (rumination)	Circuits Physiology Behavior
Langenecker et al. (2022)	US	Cohort study	MDD, BD	PVS	Reward responsiveness	Circuits Physiology Behavior Self-report
MacNamara et al. (2017)	US	Case control study	MDD, AD	NVS	Threat processing	Circuits Physiology Behavior
McTeague et al. (2020)	US	Meta-analysis	MDD, BD, AD	PVS, NVS	PVS NVS functioning (emotional processing)	Circuits
Medeiros et al. (2020)	US	RCT study	MDD	PVS, NVS	Reward responsiveness Reward learning Reward valuation Acute threat (fear) Potential threat (anxiety) Sustained threat Loss Frustrative nonreward	Molecules Self-report
Nakonezny et al. (2015)	US	Validity study	MDD	PVS	Anhedonia (reward responsiveness)	Self-report
Nusslock et al. (2015)	US	Literature review	MDD, BD, AD	PVS	Reward valuation (approach motivation)	Physiology
Nusslock and Alloy (2017)	US	Literature review	MDD, BD	PVS	Reward processing Reward valuation (approach motivation) Anhedonia	Circuits Behavior
Olino et al. (2018)	US	Cohort study	MDD^b^	PVS	Anhedonia Reward sensitivity Positive emotionality (reward responsiveness, and reward valuation)	Self-report
Paulus et al. (2017)	US	Cohort study	MDD, AD	PVS, NVS	PVS NVS functioning	Behavior Self-report
Peng et al. (2021)	US	Cohort study	MDD, AD	PVS, NVS	PVS NVS functioning	Circuits Physiology Behavior Self-report
Ross et al. (2017)	US	Literature review	MDD	NVS	Sustained threat (chronic stress)	Molecules Cells Circuits Physiology Behavior
Sambuco et al. (2020)	US	Cohort study	MDD, AD	NVS	Sustained threat	Circuits Physiology Behavior Self-report
Savage et al. (2017)	US	Literature review	AD	NVS	Acute threat (fear) Potential threat (distress) Sustained threat	Genes Molecules Circuits Physiology Behavior
Silveira and Kauer-Sant'Anna (2015)	Brazil	Systematic review	BD	NVS	Loss (rumination)	Self-report
Swope et al. (2020)	US	Cohort study	MDD^c^	PVS	Reward responsiveness Reward learning Reward valuation	Self-report
Taylor et al. (2022)	US	Literature review	MDD	PVS	PVS functioning	Cells Circuits Physiology Behavior Self-report
Terbeck et al. (2015)	UK	Literature review	MDD, AD	PVS, NVS	PVS NVS functioning	Molecules
Toups et al. (2017)	US	Cohort study	MDD	PVS	PV symptoms	Behavior Self-report
Trøstheim et al. (2020)	Norway	Meta-analysis	MDD, BD	PVS	Anhedonia (reward learning and reward valuation)	Self-report
Vaidyanathan et al. (2012)	US	Literature review	MDD, AD	NVS	Threat processing: Acute (fear) Potential (anxiety) Sustained threat	Physiology
Wenzel et al. (2022)	US	Cohort study	MDD, AD	PVS, NVS	Reward valuation Potential threat (anxiety)	Self-report
Woody and Gibb (2015)	US	Literature review	MDD	NVS	Loss (rumination)	Genes Molecules Circuits Physiology Behavior Self-report

Included sources and characteristics in alphabetical order. RDoC terms: NVS, Negative Valence Systems; PV, Positive valence; PVS, Positive Valence Systems. Origin: UK, United Kingdom; US, United States. Disorder: AD, Anxiety Disorder; BD, Bipolar Disorder; MDD, Major Depressive Disorder. Study design: RCT, Randomized Controlled Trial.

a All articles were written and published in English.

b For clarity, the specific diagnostic subtypes are listed in Tables 3–8.

c Depressive symptoms in other mental disorder or healthy population.

**Table 3:** Positive Valence Systems (PVS) primary articles.

**References**				**Units of analysis**							**PVS**				**Elements/paradigms^a^**	**Key findings**
	**Depressive disorders**	**Bipolar disorders**	**Anxiety disorders**	**Genes**	**Molecules**	**Cells**	**Circuits**	**Physiology**	**Behavior**	**Self-report**	**Domain level**	**Reward responsiveness**	**Reward learning**	**Rewardvaluation**		
Ethridge et al. (2021)	x							x		x	x				Elements: SCID-5-CV, MINI Kid, PDS, UCLA LSI; EEG recording of reward-related neural responses during doors task. Aim: examine influence of pubertal development on familial transmission of PVS functioning in mother-daughter dyads with and without maternal history of depression	Association between mothers' and daughters' reward processing was moderated by daughters' pubertal development with dyads becoming more similar at more advanced stages of puberty; maternal history of depression was linked to reduced reward response at more advanced stages of puberty → relationship between familial psychopathology risk and PVS functioning may change over the course of adolescent development
Alexopoulos et al. (2015)	x								x	x			x	x	Elements: SCID-R, WHO-DAS, HAM-D, MMSE, HVLT, NEO; Engage therapy. Constructs: reward exposure (reward learning, reward valuation); barriers of reward exposure [negativity bias (loss), apathy (arousal), and emotional dysregulation (cognitive control)]. Aim: proof of concept study of the efficacy of Engage as a streamlined RDoC psychotherapy	Psychotherapy using neurobiological constructs to identify and use behavioral strategies to promote engagement in meaningful, rewarding activities, thereby increasing reward exposure → preliminary evidence suggests that Engage as the first RDoC-based neuroscience-driven psychotherapeutic intervention constitutes an efficacious approach to the treatment of late-life depression
Alexopoulos et al. (2016)	x								x	x			x	x	Elements: Engage therapy (9-week treatment for late-life depression); training of reward exposure (engagement in meaningful, rewarding activities); SCID-R, WHO-DAS, HAM-D, BADS (measurement points at baseline, and after 6, 9 (end of treatment), and 36 weeks), MMSE, Stroop CWIT, response inhibition test, DRS-IP, HVLT-R. Constructs: reward learning, reward valuation	Changes in behavioral activation (BA) led to improvement of late-life MDD symptoms during Engage treatment and follow-up: both BA and late-life MDD symptoms significantly changed; at each observation period, change in BA and time predicted MDD severity
Toups et al. (2017)	x								x	x	x				Elements: 12-week treatment with Exercise Augmentation for Depression (TREAD) study; SHAPS, MEI, QIDS. Domain level: PV symptoms	SHAPS and MEI scores significantly improved with exercise; MEI score change was a significant moderator and mediator of exercise in MDD → PV symptoms improve with exercise treatment for depression; PV symptoms: motivation and energy more clinically relevant than anhedonia
Nakonezny et al. (2015)	x									x		x			Element: SHAPS, HAM-D 17, IDS-C 20, IDS-SR 30, QIDS-C-16, QIDS-SR-30, QLES-Q. Subconstruct: anhedonia (hedonic ⇄ anhedonic)	PCA confirmed a unidimensional (hedonic experience) factor structure and the SHAPS as a reliable and valid instrument (pos. associations to psychometric scales) to examine hedonic experience (PV) in MDD outpatients
Olino et al. (2018)	x ^b^									x	x	x		x	Elements: PAS, SAS, FCPS, SHAPS, TEPS, PANAS (PA only), BIS/BAS, CES-D (PA only), BFI, PROMIS-Dep. Constructs: anhedonia, reward sensitivity, and positive emotionality (PE)	Associations between latent factors (sociability, PE, assertiveness, pleasure seeking, BA) and self-reported depressive symptoms: (1) EFA solution: all factors negatively associated (PE strongest); (2) Bifactor solution: only two specific and the general factor negatively associated (again, PE strongest) → results help to understand the contribution of the PVS to depressive psychopathology
Swope et al. (2020)	x^b^									x		x	x	x	Elements: ACI, BAARS-IV (ADHD), DASS-21 depression subscale, VHS, SBI anticipating subscale, TEPS-ANT, BIS/BAS drive subscale, SBI savoring the moment subscale, DPES, RPA positive rumination subscale. Aim: situate Sluggish Cognitive Tempo (SCT) within RDoC framework by investigating relationship with PVS components	SCT within RDoC PVS components: SCT was associated with increased reward valuation and expectancy but reduced willingness to work for rewarding experiences; no unique relationship between SCT and initial/sustained reward response; depressive symptoms linked to increased reward valuation and reduced expectancy, willingness to work, initial and sustained reward response → both SCT and depressive symptoms found to be uniquely related to PVS while controlling for demographic factors and co-occurring psychopathology
Khazanov et al. (2020)	x									x		x	x	x	Element: PVSS-21. (Sub-)constructs: reward processing (anticipation, responsiveness, learning, valuation, satiation, and anhedonia)	PVSS-21 showed strong internal consistency, retest reliability, and factorial validity; it showed a stronger correlation with reward sensitivity than punishment sensitivity, PA than NA, and depression than anxiety; PVSS-21 scores distinguished depressed from non-depressed and predicted anhedonia severity, even after adjusting for depression; PVSS-21 has potential in improving our understanding of reward-related abnormalities in depression and other disorders
Gruber et al. (2015)	x	x						x		x	x	x			Elements: ambulatory psycho-physiological measurement (6-day consecutive period): HRV-HF (mean level, intra-individual variability, sympathetic activity, somatic movement → positive emotional disturbances), cardiovascular arousal (HR), sympathetic nervous system activity (skin temperature); GSM, mDES (state/trait PA). Constructs: positive affectivity (state PA, trait PA), reward responsiveness (initial response to reward)	Consistent with the HRV-HF instability hypothesis: BD exhibited higher HRV-HF instability compared to both MDD and CG (due to subsyndromal remitted manic symptoms); results support models of PVS disturbances and underlying psychophysiological mechanisms
Langenecker et al. (2022)	x	x					x	x	x	x		x			Disorder: MDD, BD, Mood disorder not otherwise specified, HC. Elements: resting-state fMRI, parametric Go/NoGo test, monetary incentive delay task. (Sub-)constructs: reward responsiveness, inhibitory control. Aim: identify potential disease connectome edges as biomarkers of risk for mood disorder recurrence in participants with currently remitted MDD/BD and HC	No behavioral performance differences between mood disorder patients and HC in reward responsiveness and inhibitory control; differences in reward responsiveness reflected as differences in negative edges in the ventral attention/salience and emotion network; no overlap in edges related to diagnostic group membership and reward responsiveness connectomic profiles → no evidence of disrupted reward responsiveness in remitted mood disorders → role of reward responsiveness as a proximal marker of acute MDD/BD symptoms

RDoC terms: NA, Negative Affect; NV, Negative Valence; PA, Positive Affect(ivity); PV, Positive Valence; PE, Positive Emotion; PVS, Positive Valence Systems; RDoC, Research Domain Criteria. Disorder: ADHD, Attention Deficit Hyperactivity Disorder; BD, Bipolar Disorder; MDD, Major Depressive Disorder; SZ, Schizophrenia. Physiology: EEG, Electroencephalography; fMRI, functional Magnetic Resonance Imaging; HR, Heart Rate; HRV-HF, High-frequency Heart Rate Variability; GSM, Gross Somatic Movement; PET, Positron Emission Tomography. Behavior: BA, Behavioral Activation; SCT, Sluggish Cognitive Tempo; Stroop CWIT, Stroop Color Word Interference Test; TREAD, Treatment with Exercise Augmentation for Depression. Self-report/Interview: BADS, Behavioral Activation for Depression Scale; BAARS-IV, Barkley Adult ADHD Rating Scale-IV; BFI, Big Five Inventory; BIS/BAS, Behavioral Inhibition and Behavioral Activation Scales; CES-D, Center for Epidemiologic Studies Depression Scale; DASS-21, Depression Anxiety Stress Scales-21; mDES, modified Differential Emotions Scale; DPES, Dispositional Positive Emotion Scale; DRS-IP, Dementia Rating Scale, Initiation/Perseveration Scale; FCPS, Fawcett–Clark Pleasure Scale; GAF, Global Assessment of Functioning; HAM-D, Hamilton Depression Rating Scale; HVLT, Hopkins Verbal Learning Test; IDS-C/SR, Inventory of Depressive Symptomatology—Clinician rated/Self-rated; MEI, Motivation and Energy Inventory; MINI Kid, Mini-International Neuropsychiatric Interview for Children and Adolescents; MMSE, Mini-Mental State Examination; MPQ-BF, Multidimensional Personality Questionnaire—Brief Form; NEO, Neuroticism-Extroversion-Openness Personality Inventory; PANAS, Positive and Negative Affect Scale; PANSS, Positive and Negative Syndrome Scale; PAS, Physical Anhedonia Scale; PDS, Pubertal Development Scale; PROMIS-Dep, Patient-Reported Outcomes Measurement Information System—Depression; PVSS-21, Positive Valence Systems Scale; QIDS-C/SR, Quick Inventory of Depression Symptomatology—Clinician rated/Self-rated; QLES-Q, Quality of Life Interview for Enjoyment, and Satisfaction Questionnaire; RPA, Response to Positive Affect Scale; SAS, Social Anhedonia Scale; SBI, Savoring Beliefs Inventory; SCID-5-CV, Structured Clinical Interview for DSM−5 Disorders Clinician Version; SCID-R, Structured Clinical Interview for DSM-IV; SHAPS, Snaith–Hamilton Pleasure Scale; TEPS, Temporal Experience of Pleasure Scale; TEPS-ANT, Temporal Experience of Pleasure Scale Anticipatory Subscale; UCLA LSI, University of Los Angeles, Life Stress Interview; VHS, Valuing Happiness Scale; WHO-DAS, World Health Organization Disability Assessment Schedule; YMRS, Young Mania Rating Scale. Methods: CG, Control Group; EFA, Exploratory Factor Analysis; HC, Healthy Controls; PCA, Principal Component Analysis.

a Specific disorder codes were added if this provides additional information.

b Depressive symptoms in other mental disorder or healthy population.

**Table 4:** Positive Valence Systems (PVS) reviews.

**References**				**Units of analysis**							**PVS**				**Main aim of review^a^**	**Key findings**
	**Depressive disorders**	**Bipolar disorders**	**Anxiety disorders**	**Genes**	**Molecules**	**Cells**	**Circuits**	**Physiology**	**Behavior**	**Self-report**	**Domain level**	**Reward responsiveness**	**Reward learning**	**Rewardvaluation**		
Taylor et al. (2022)	x					x	x	x	x	x	x				Disorder: LLD. Elements: dopaminergic system, inflammation markers, neuroimaging. Constructs: reward processing, cognitive systems, sensorimotor systems. Aim: develop a model for the relationship between dopaminergic dysfunction and LLD	Age-related reduction in dopamine system signaling contributes to deficits in positive valence systems (reflected in higher effort cost as well as reduced motivation and reward learning) that combine and interact with impairments in cognitive and sensorimotor systems to increase vulnerability to LLD
Baskin-Sommers and Foti (2015)	x					x	x	x				x	x	x	Elements: GABA, OFC, ACC, d/rACC, NAc, ventral tegmentum, ventral pallidum, amygdala; EEG, fMRI. Constructs: initial responsiveness to reward (liking), reward valuation (approach motivation, wanting), reward learning (habit formation). Aim: overview of preclinical, electro-physiological, and neuroimaging literature on reward processing from a transdiagnostic, multidimensional perspective	Individual differences in reward sensitivity associated with risk for substance abuse and depression: MDD: blunted reactivity to monetary reward in striatum, incl. bilateral putamen, caudate, and NAc; deficit in striatal activation to types of pleasant stimuli; impaired reward learning associated with blunted activity within the NAc, dACC, and rACC
Barch et al. (2016)	x						x		x			x	x	x	Elements: circuit-behavioral, ERP and fMRI measures. Constructs: initial responsiveness to reward, reward anticipation or expectancy, incentive or reinforcement learning, effort valuation, action selection. Aim: review of impairments in motivational and hedonic constructs in individuals with psychosis vs. with depressive pathology	Differences of reward-related and hedonic deficits associated with psychosis vs. depression; (anhedonic) MDD: hedonic impairments → these deficits may trigger other impairments (anticipation, learning, effort, and action selection); hedonic impairments associated with alterations in dopamine and/or opioid signaling in the striatum (relatively intact hedonic processing in psychosis, but impaired reward learning and action selection)
Fettes et al. (2017)	x						x	x					x	x	Elements: structural and functional neuroimaging (VBM, fMRI, and PET), neurostimulatory techniques (DBS, ECT, rTMS, and tDCS). Constructs: reward-guided learning, reward valuation (reappraisal). Aim: review role of disturbances in cortico-striatal-thalamic loop circuits of the OFC (mOFC and lOFC) in MDD, OCD, and SUD	OFC-striatal circuits play a key role in reward valuation, affect regulation, and decision-making; dysfunction in these circuits is associated with OCD, MDD, and SUD symptomatology; abnormal activity in mOFC or lOFC-striatal pathways is amenable to intervention by invasive as well as non-invasive brain stimulation techniques → neurostimulation inventions may have the potential selectively modulate psychiatric symptoms related to OFC dysfunction
Nusslock et al. (2015)	x	x	x					x						x	Element: EEG. Construct: Reward valuation (approach motivation). Aim: relationship between relative left frontal EEG activity and mood and anxiety related symptoms → approach–withdrawal motivational model of frontal EEG asymmetry	Greater relative left frontal EEG activity (increased approach motivation → BD/manic symptoms) and decreased relative left frontal EEG activity (decreased approach motivation or increased withdrawal tendencies → MDD/anhedonia); specific symptom clusters of depression (anhedonia), hypomania/mania (excessive approach motivation), and anxiety (apprehension vs. anxious arousal)
Nusslock and Alloy (2017)	x	x					x		x		x		x	x	Element: circuit-behavioral. (Sub-)constructs: reward valuation (approach motivation), anhedonia. Aim: relationship between reward processing and mood-related symptoms	MDD and BD have distinct patters of reward processing and reward-related brain activity; anhedonia in MDD is characterized by reward hyposensitivity and decreased approach motivation; reward hypersensitivity and elevated approach motivation relates to (hypo-)manic symptoms in BD
Trøstheim et al. (2020) [Meta-Analysis]	x	x								x				x	Disorder: (current/past/remitted) MDD, BD, HC. Element: SHAPS (assessed at baseline or in a no-treatment condition). Subconstruct: Anhedonia. Aim: generate and compare reference values for anhedonia levels in adults with and without mental illness	Patients scored higher on the SHAPS than HC; MDD higher than all other patient groups (remitted MDD within the healthy range) → anhedonia in MDD affects multiple pleasure domains; less effects for other disorders

RDoC terms: PVS, Positive Valence Systems; RDoC, Research Domain Criteria. Disorder: BD, Bipolar Disorder; MDD, Major Depressive Disorder; LLD, Late-life Depression; OCD, Obsessive-compulsive Disorder; SUD, Substance Use Disorder. Circuit: ACC, Anterior Cingulate Cortex; dACC, dorsal Anterior Cingulate Cortex; rACC, rostral Anterior Cingulate Cortex; OFC, Orbitofrontal Cortex; medial Orbitofrontal Cortex; l/mOFC, lateral/medial Orbitofrontal Cortex; NAc, Nucleus Accumbens. Physiology: DBS, Deep Brain Stimulation; tDCS, transcranial Direct Current Stimulation; ECT, Electroconvulsive Therapy; EEG, Electroencephalography; ERP, Event-Related Potential; fMRI, functional Magnetic Resonance Imaging; PET, Positron Emission Tomography; rTMS, Repetitive Transcranial Magnetic Stimulation; VBM, Voxel-Based Morphometry. Self-report/Interview: HAM-D, Hamilton Depression Rating Scale; SHAPS, Snaith–Hamilton Pleasure Scale; WHO-DAS, World Health Organization Disability Assessment Schedule. Methods: HC, Healthy Controls.

a Specific disorder codes were added if this provides additional information.

**Table 5:** Negative Valence Systems (NVS) primary articles.

**References**				**Units of Analysis**							**NVS**					**Elements/paradigms^a^**	**Key findings**
	**Depressive disorders**	**Bipolar disorders**	**Anxiety disorders**	**Genes**	**Molecules**	**Cells**	**Circuits**	**Physiology**	**Behavior**	**Self-report**	**Acutethreat**	**Potential threat**	**Sustained threat**	**Loss**	**Frustrative nonreward**		
Ellingson et al. (2016)	x			x						x		x				Elements: MPQ stress reactivity (SR) and control (CON) subscales, CIDI (AUD section), AUDADIS-IV (modified version for MDD assessment). Aim: examine whether covariation between MDD and AUD can be traced back to phenotypic, genetic, and environmental variance in NE and behavioral control	SR and CON explained 70% of the genetic and 20% of the environmental covariation between MDD and AUD (sample of same-sex DZ and MZ twins), with trait measures of behavioral control accounting for unique covariation between MDD and AUD beyond what was explained by NE → first study to show role of NE and behavioral control as modulators of risk for the co-occurrence of MDD and AUD via genetic and environmental factors
Gunzler et al. (2020)	x									x				x		Element: PHQ-9. Construct: Loss (anhedonia, guilt, and self-harm). Aim: use FA and qualitative analysis to identify depressive phenotypes by mapping PHQ items along RDoC domains	Four depressive phenotypes: NVS and externalizing (anhedonia, depression), NVS and internalizing (depression, guilt, and self-harm), ARS (sleep, fatigue, and appetite), and CS and SmS (concentration, psychomotor)
MacNamara et al. (2017)	x		x				x	x	x		x					Disorder: MDD, AD (GAD, SAD). Elements: fMRI; emotional face-matching task during processing of affective scenes and faces (fearful, angry, happy and geometric shapes); bilateral insula, ACC, MCC and dlPFC. Construct: threat processing	Transdiagnostic anxiety and depressive symptomatology marked by activation in paralimbic, cingulate, and lateral prefrontal regions in response to angry faces; lateral prefrontal activation may be affected differently by symptoms of anxiety and depression; shared neural dysfunction in threat processing in patients with GAD, SAD, and MDD → varies with symptom severity
Sambuco et al. (2020)	x		x				x	x	x	x			x			Disorder: MDD, AD (SAD, GAD, SPD, and PD), HC. Elements: fMRI; rapid serial visual presentation/emotional scene processing (IAPS); BDI-II, STAI, IIRS, MASQ, PAS, and PDS. Construct: sustained threat (trauma severity). Primary analyses: functional brain activity in the amygdala, inferotemporal, and occipital visual cortex. Additional: BOLD activity association to individual trauma factor	Emotional reactivity: amygdala and ventral visual cortex (BOLD) activity enhanced when watching emotional arousing scenes; changes during emotional processing predicted self-reported experienced trauma, PTSD-like symptoms and associated functional impairment; highest (lowest) trauma scores ↔ smallest (largest) changes in BOLD; experience of a life- threatening event associated with reduced functional limbic-visual activity. Experienced trauma may be a common underlying factor contributing to the development of psychopathology in patients with various anxiety and mood disorders
Cochran et al. (2020)	x		x							x		x		x	x	Disorders: MDD (MDD, DD-NOS), AD (GAD, PD, SAD). Elements: STAI, HAM-D, HAM-A, EPDS, BDI, PSS, and PSQI. Aim: establish transdiagnostic framework for understanding depressive and related symptoms during pregnancy and postpartum. Method: FA (bifactor model)	FA bifactor model with six transdiagnostic factors (loss, potential threat, Frustrative nonreward, sleep-wakefulness, somatic, and coping) and general factor showed good fit, but model components needed to vary across perinatal period → overall coherence of factor structures for depressive symptomatology, yet importance of certain symptoms as pathology markers changed between earlier pregnancy and later postpartum → symptoms may need to be viewed in connection with specific life phases within as well as beyond the perinatal context
Guineau et al. (2022)	x		x							x				x		Elements: SCID-IV-RV, MATE, DIVA, NIDA, anhedonia items of the OQ-45-2, IDS-SR, ASI, CAARS-S, AQ-50. Method: graphical Least Absolute Shrinkage and Selection Operator (LASSO) network	Anhedonia severity predicted severity of depressive and (to a lesser extent) anxious, ADHD, and ASD symptoms; reverse influences on anhedonia severity existed but were less pronounced → role of anhedonia as a transdiagnostic feature of psychopathology
Lang et al. (2016)	x		x				x	x	x	x	x					Disorders: MDD, AD (PD, AG, GAD, SAD, SPD, and AD-NOS). Paradigm: Emotional imagery. Elements: HR reactivity, skin conductance level, facial EMG, blink-response magnitude during ideographic fear imagery with startle probes; Anxiety Sensitivity Index, BDI-II, MASQ subscales, STAI-trait, STAXI-trait, FSS, IIRS, subscales of SRRS, and 17-item checklist of early life stressor occurrence	Self-report: PCA resulted in three factors negative affectivity/general distress, anxious/hyperarousal and cumulative life stress. Composite index of startle reflex and heart rate reactivity during idiographic fear imagery for each patient was translated into a defensive dimension defined by ranking patients from most defensively reactive to least reactive → paralleled by diminishing reactivity in electrodermal and facial EMG reactions across this defensive dimension. Only PCA factor negative affectivity/general distress showed association to defensive dimension—as distress levels increased, defensive reactivity decreased. → each principal diagnosis was represented in every quintile → within-diagnosis heterogeneity regarding defensive reactivity
Lang et al. (2018)	x		x					x		x	x					Disorder: MDD, AD (SPD, SAD, PD, PD/AG, GAD, and other AD). Elements: event-related potential (acoustic startle probes), EEG; BDI-II, MASQ subscales, STAI-trait, IIRS, and SUDS	Reduced positive amplitude of centro-parietal startle-evoked event-related potential were related to higher scores of depression/anxiety, increased life dysfunction, greater co-morbidity and disease severity and less favorable prognosis → startle reaction predicted severity and extent of psychopathology

RDoC terms: ARS, Arousal and Regulatory Systems; CS, Cognitive Systems; NE, Negative Emotionality; NVS, Negative Valence Systems; RDoC, Research Domain Criteria; SmS, Sensorimotor Systems. Disorder: ADHD, Attention Deficit Hyperactivity Disorder; AD-NOS, Anxiety Disorder Not Otherwise Specified; AG, Agoraphobia; ASD, Autism Spectrum Disorder; AUD, Alcohol Use Disorder; DD-NOS, Depressive Disorder Not Otherwise Specified; GAD, General Anxiety Disorder; MDD, Major Depressive Disorder; PD, Panic Disorder; PD/AG, Panic Disorder with Agoraphobia; PTSD, Post-Traumatic Stress Disorder; SAD, Social Anxiety Disorder; SPD, Specific Phobic Disorder. Genes: DZ/MZ, Di-/Monozygotic. Circuit: ACC, Anterior Cingulate Cortex; BOLD-activity, Blood-oxygen-level dependent activity; MCC, Midcingulate Cortex; PFC, Prefrontal Cortex; dlPFC, dorsolateral Prefrontal Cortex. Physiology: EEG, Electroencephalography; EMG, Electromyography; HR, Heart rate; fMRI, functional Magnet Resonance Imaging. Behavior: IAPS, International Affective Picture Systems. Self-report/Interview: AQ-50, Autism Spectrum Quotient; ASI, Anxiety Sensitivity Index; AUDADIS-IV, Alcohol Use Disorder and Associated Disabilities Interview Schedule-IV; BDI/BDI-II, Beck Depression Inventory; CAARS-S, Conners' Adult ADHD Rating Scale; CIDI, Composite International Diagnostic Interview; DIVA, Diagnostic Interview for ADHD in adults; EPDS, Edinburgh Postnatal Depression Scale; FSS, Fear Survey Schedule; HAM-A, Hamilton Anxiety Rating Scale; HAM-D, Hamilton Depression Rating Scale; IDS-SR, Inventory of Depressive Symptomatology Self-Report; IIRS, Illness Intrusiveness Rating Scale; MASQ, Mood and Anxiety Symptoms Questionnaire; MATE, Structured Interview Measurements in the Addictions for Triage and Evaluation; MPQ, Multidimensional Personality Questionnaire; NIDA, Dutch Interview for Autism Spectrum Disorders in Adults; OQ-45-2, Outcome Questionnaire; PAS, Panic and Agoraphobia Scale; PDS, Posttraumatic Diagnostic Scale; PHQ-9, Patient Health Questionnaire-9; PSQI, Pittsburg Sleep Quality Index; PSS, Perceived Stress Scale; SCID-IV-RV, Structured Clinical Interview for DSM-IV Axis I Disorders; SRRS, Social Readjustment Rating Scale; STAI-trait, State-Trait Anxiety Inventory; STAXI-trait, State-Trait Anger Expression Inventory; SUDS, Subjective Units of Distress Scale. Methods: FA, Factor Analysis; HC, Healthy Controls; LASSO, Least Absolute Shrinkage and Selection Operator; PCA, Principal Component Analysis.

a Specific disorder codes were added if this provides additional information.

**Table 6:** Negative Valence Systems (NVS) reviews.

**References**				**Units of analysis**							**NVS**					**Main aim of review^a^**	**Key findings**
	**Depressive disorders**	**Bipolar disorders**	**Anxiety disorders**	**Genes**	**Molecules**	**Cells**	**Circuits**	**Physiology**	**Behavior**	**Self-report**	**Acute threat**	**Potential threat**	**Sustained threat**	**Loss**	**Frustrative nonreward**		
Woody and Gibb (2015)	x			x	x		x	x	x	x				x		Elements: Genes: MAOA, COMT, DAT1, 5-HTRs. Molecules: downregulation of GCR, upregulation of CRH, estrogens, androgens, oxytocin, vasopressin, inflammatory molecules. Circuits: sustained amygdala reactivity, decreased dlPFC Recruitment, decreased vmPFC (incl. rostral cingulate), increased insula activation, increased PCC activity, decreased R Parietal, PVN, hippocampus, OFC, habit systems (striatum/caudate/accumbens), increased DMN activity, dysregulated reward circuitry). Physiology: ANS, HPA, neuroimmune dysregulation, prolonged psychophysiological reactivity. Behavior: rumination, withdrawal, worry, crying, sadness, loss-relevant recall bias, shame, attentional bias to negative valenced information, guilt, morbid thoughts, psychomotor retardation, anhedonia, increased self-focus, deficits in executive function (e.g., impaired sustained attention), loss of drive (sleep, appetite, libido), amotivation. Self-report: change in attributional style, hopelessness. Aim: depression research with focus on loss (rumination) within RDoC framework	Circuitry: disruption of cortico-limbic circuitry; increased activity in default mode network. Genes: regulating neurotransmission of monoamines. Molecular: e.g., sex hormones. Physiology: pupil dilation. Behavior: heterogenous list of features. Self-report: attributional style; hopelessness → summary: rumination dynamic processes influencing neurodevelopmental progression of MDD; imaging studies mostly focused on current MDD; loss as a RDoC construct; loss in environmental and developmental contexts; research of rumination as an example of RDoC research
Ross et al. (2017)	x				x	x	x	x	x				x			Disorder: (anxious) MDD. Elements: inflammation processes, alterations in protein expression; neurocircuitry alterations (BNST, amygdala); ERN; HPA axis dysfunction; reduced reactivity to passive stimuli, increased activity to aversive stimuli; clinical profiles. Construct: sustained threat (chronic stress)	Anxious MDD: increased risk for treatment-resistance to standard antidepressants, increased morbidity risk; review of the impact of sustained threat (chronic stress: childhood trauma, physical illness) on (anxious) MDD through pathological changes to molecules, cells, neurocircuitry, physiology, and behavior
Gibb et al. (2016)	x		x	x			x	x	x				x	x		Elements: dot probe task (RT indices), Posner spatial cueing task, Stroop task, eye-tracking/passive viewing task, ERPs. Constructs: sustained threat, loss (attention bias to specific stimuli for both). Aim: review findings regarding genetic influence on attentional biases in clinical and non-clinical populations (twin and candidate gene studies)	Review of approaches to assessing biased attention to emotional stimuli with links to psychopathology and neural influences: MDD and AD patients exhibit disorder-specific alterations in attentional bias for affectively-salient stimuli; twin and candidate gene studies show robust genetic influence on attentional bias; development of attentional bias is influenced by specific environmental factors that can interact with genes related to HPA axis reactivity
Vaidyanathan et al. (2012)	x		x					x			x	x	x			Disorder: MDD, (phobic/non phobic) AD (SPD, SAD, PD, PD/AG, and GAD). Elements: ERN, startle blink reflex. Constructs: threat processing. Aim: physiological measures among disorders entailing salient anxiety or depressive symptomatology	Phobia: normal-range ERN, increased startle to unpleasant stimuli; non-phobic AD: increased ERN, increased startle across all types of emotional stimuli and increased baseline startle; MDD: patterns of response for startle and ERN appear to vary as a function of severity and distinct symptomatology; fear, anxiety and depression are distinguishable (by ERN and startle results) constructs
Klumpp and Shankman (2018)	x		x					x			x	x	x			Disorder: MDD, AD (PD, SAD, and GAD). Elements: ERP, ERN, startle-blink, negative processing bias. Construct: threat processing. Aim: how can ERP and startle studies inform the role of chronometry in anxiety and depression	ERP: individuals with internalizing psychopathologies show transdiagnostic abnormalities in early stages of processing; startle reactivity: fear-based disorders (PD, SAD) can be distinguished from other AD (GAD) and different internalizing phenotypes show different patterns of habituation
Boecker and Pauli (2019)	x		x					x			x	x	x			Disorder: MDD, AD (PD, PD/AG, SPD, SAD, GAD). Elements: EMG of the musculus orbicularis oculi, affective startle modulation (ASM). Construct: threat processing. Aim: review findings regarding ASM anomalies across psychiatric categories; motivational priming hypothesis	Different psychopathologies were related to specific changes in startle potentiation/attenuation, anomalies in only one motivational system: increased startle potentiation to unpleasant stimuli (AD); general hyporeactivity to affective stimuli (MDD); increased vs. decreased startle responses to disorder-specific stimuli (SPD)
Savage et al. (2017)			x	x	x		x	x	x		x	x	x			Constructs: acute threat (fear), potential threat, sustained threat (distress). Aim: review of genetic epidemiological data (twin studies, heritability) and molecular genetic findings for NVS phenotypic measures	Molecular genetic basis of NVS phenotypes (early stages); little research (attentional bias, peripheral physiology, or brain-based measures of threat response); most studies with small number of genes selected for putative association to AD; current NVS constructs may be too broad (including constructs with little overlap, e.g., threat vs. loss) for genetic analyses
Hamm et al. (2016)			x	x				x	x		x	x	x			Disorder: AD (PD/AG). Elements: BAT; heart rate, skin conductance, startle blink; MAOA-uVNTR, 5-HTR1A. Construct: threat processing	Panic attacks (strike defense): fear reaction to acute threat with desire to actively avoid or flee when internal threat stimuli are impending, related to genetic modulators within serotonergic system; anxious apprehension (postencounter defense): related to general distress and depressive mood, as to genetic modulations within HPA axis
Silveira and Kauer-Sant'Anna (2015) [systematic review]		x								x				x		Objective: systematic review of rumination in BD; inclusion criteria: studies involving at least one validated scale for the assessment of rumination (reviews were excluded)	Rumination is present in all BD phases; associated with symptoms of depression, anxiety, hypomania; no research on neurobiological findings; independent of mood state; negative impact on cognitive and executive functions (inhibitory control); rumination in response to both PA and NA possible; lack of neurobiological research

RDoC terms: NA, Negative affect; NVS, Negative Valence Systems; PA, Positive affect; RDOC, Research Domain Criteria. Disorder: AD, Anxiety Disorder; BD, Bipolar Disorder; GAD, General Anxiety Disorder; MDD, Major Depression Disorder; PD, Panic Disorder; PD/AG, Panic Disorder with Agoraphobia; SAD, Social Anxiety Disorder; SPD, Specific Phobic Disorder. Genetic: COMT, Catechol-O-Methyltransferase; DAT-1, Dopamine Active Transporter-1; 5-HTRs, 5-Hydroxytryptamine Receptors; 5-HTR1A, 5-Hydroxytryptamine Receptor 1A; MAOA, Monoamine Oxidase; MAOA-uVNTR, Monoamine Oxidase A—Upstream Variable Number of Tandem Repeats. Molecules: CRH, Corticotropin-releasing hormone GCR, Glucocorticoid receptor. Circuit: BNST, Bed Nucleus of the Stria Terminalis; DMN, Default Mode Network; OFC, Orbitofrontal Cortex; PPC, Posterior Cingulate Cortex; dlPFC, dorsolateral Prefrontal Cortex; vmPFC, ventromedial Prefrontal Cortex; PVN, Paraventricular Nucleus. Physiology: ANS, Autonomic Nervous System; ASM, Affective Startle Modulation; EMG, Electromyography; ERN, Error-related Negativity; ERP, Event-related Potential; HPA axis, Hypothalamic–Pituitary–Adrenal axis. Behavior: BAT, Behavioral Avoidance Task; RT, Reaction Time.

a Specific disorder codes were added if this provides additional information.

**Table 7:** Cross-domain primary articles.

**References**				**Units of analysis**							**PVS**				**NVS**						**Elements/paradigms^a^**	**Key findings**
	**Depressive disorders**	**Bipolar disorders**	**Anxiety disorders**	**Genes**	**Molecules**	**Cells**	**Circuits**	**Physiology**	**Behavior**	**Self-report**	**Domain level**	**Reward responsiveness**	**Reward learning**	**Reward valuation**	**Domain level**	**Acute threat**	**Potential threat**	**Sustained threat**	**Loss**	**Frustrative nonreward**		
Medeiros et al. (2020)	x				x					x		x	x	x		x	x	x	x	x	Elements: immunomarkers (CRP, IFN-γ, Il-1β, Il-2, Il-4, Il-6, Il-8, Il-10, and TNF-α), IDS-C30 and QIDS-C16, HAM-D, CAST, WSAS, CPFQ, PDSQ, self-administered comorbidity questionnaire (medical comorbidities)	PV and NV symptom scores in a MDD-sample were linked to different clinical characteristics: (1) PV symptom scores (impaired motivation etc.) positively correlated with female gender, older age, higher impairment, the level of three pro- and one anti-inflammatory immuno-markers; (2) NV symptom scores (anxiety and interpersonal sensitivity) correlated with younger age, anxious comorbidities, and symptom load and were negatively associated with only one proinflammatory immuno-marker; antidepressants were more effective on PV symptoms than NV symptoms
Peng et al. (2021)	x		x				x	x	x	x	x				x						Elements: MID [reward p(e.g., ventral striatum, insula, NAcc, caudate head) and loss processing (e.g., anterior insula)], fear conditioning fMRI task (vmPFC, dmPFC, sgACC, dACC, left/right anterior insula, ventral hippocampus, and amygdala), HR/SCR while rating emotional pictures and while performing MTPT; OASIS, PHQ-9, PANAS, GAD-7, MASQ, BIS/BAS, SPSRQ, TEPS, AcSEAS. Domain level: PVS functioning, NVS functioning	Group FA could not identify any latent variables that could explain variance across tasks; instead, variance was best explained by individual variables within each task (*Post hoc* analyses: (1) small effect sizes between latent variables from fMRI and self-report data and (2) some latent variables not directly related to individual PVS/NVS constructs) → lack of cross-modal latent structure suggests challenges in the RDoC approach and highlights the need for more targeted approaches
Paulus et al. (2017)	x		x						x	x	x	x	x	x	x	x		x			Elements: AAT, MPDT, PANAS, MASQ, TEPS, BIS/BAS, SPSRQ, PID-5, AsSEAS, WHO-DAS, GAD-7. Latent variable analysis (PCA) underlying PVS and NVS processing in terms of symptoms and behavioral units of analysis	PCA resulted in two “meta-components”: NVS processing (NV symptoms, negative approach bias, high sustained and selective attention), PVS processing (PV symptoms, positive approach bias, slow selective or sustained attention)
Wenzel et al. (2022)	x		x							x				x			x				Disorder: perinatal MDD, perinatal AD. Elements: BIS/BAS (BIS → potential threat; BAS drive → reward valuation), IUS (potential threat), PHQ-9, GAD-7	Higher trait BIS+IUS (time stable potential threat), higher state BIS (time variant potential threat) and lower state BAS drive (reward valuation) were associated with higher depressive symptom burden; Higher trait BIS+IUS and higher state BIS was associated with higher anxiety symptom burden; Potential threat may be a transdiagnostic feature of perinatal anxiety and depression, reward valuation may be non-transdiagnostic, weaker feature of perinatal depression; Potential threat showed relevance as both a “trait-like” feature (sustained across perinatal period) and a “state-like” feature (within-variability across pregnancy)
Förstner et al. (2022)	x	x	x						x	x	x	x	x		x		x				Elements: TMT A/B, DSST; BSI-53, PANAS, WHO-DAS 2.0	A CFA four-factor latent structure (PVS, NVS, CS, SP) was validated indicating a transnosological latent structure for these domains. Well-established assessments can be used to measure RDoC constructs

RDoC terms: CS, Cognitive Systems; NV, Negative valence; NVS, Negative Valence Systems; PVS, Positive Valence Systems; RDoC, Research Domain Criteria; SP, Social Processes. Disorder: AD, Anxiety Disorder; BD, Bipolar Disorder; MDD, Major Depressive Disorder; SUD, Substance Use Disorder. Molecules: CRP, C-reactive Protein; IFN-γ, Interferon-γ Necrosis Factor; TNF-α, Tumor Necrosis Factor-α; Il, Interleukin. Circuit: dACC, dorsal Anterior Cingulate Cortex; pgACC, pregenual Anterior Cingulate Cortex; NAcc, Nucleus Accumbens; dmPFC, dorsomedial Prefrontal Cortex; vmPFC, ventromedial Prefrontal Cortex. Physiology: fMRI, functional Magnet Resonance Imaging; HR, Heart Rate; SCR, Skin Conductance Response. Behavior: AAT, Approach Avoidance Task; DSST, Digit Symbol Substitution Test; MPDT, Modified Probe Detection Task; MTPT, Mirror Tracing Persistence Task; TMT-A/B, Trail Making Test-Version A/B. Self-report/Interview: AsSEAS, Acceptance, Safety, Escape/Avoidance Scale; BIS/BAS, Behavioral Inhibition and Behavioral Activation Scales; BFNE-S, Brief Fear of Negative Evaluation Scale—Straightforward Items; BSI-53, Brief Symptom Checklist; CAST, Concise Associated Symptoms Tracking; CPFQ, Cognitive and Physical Functioning Questionnaire; GAD-7, General Anxiety Disorder; HAM-D, Hamilton Rating Scale for Depression; IDS-C30, Depressive Symptomatology-Clinician Version; IUS, Intolerance of Uncertainty Scale; MASQ, Mood and Anxiety symptom Questionnaire; MINI, Mini International Neuropsychiatric Interview; OASIS, Overall Anxiety Severity and Impairment Scale; PANAS, Positive and Negative Affect Schedule; PDSQ, Psychiatric Diagnostic Screening Questionnaire; PHQ-9, Patient Health Questionnaire-9; PID-5, Personality Inventory for DSM-5-Adult; QIDS-C, Quick Inventory of Depression Symptomatology - Clinician rated; RTQ, Repetitive Thinking Questionnaire; Shipley, Shipley Institute of living Scale—Vocabulary Test; SPSRQ, Sensitivity to Punishment and Sensitivity to Reward Questionnaire; TEPS, Temporal Experience of Pleasure Scale; WHO-DAS, World Health Organization Disability Assessment Schedule; WSAS, Work and Social Adjustment Scale. Paradigms: MID, Monetary Incentive Delay; MTPT, Mirror Tracing Persistence Task. Methods: CFA, Confirmatory Factor Analysis; FA, Factor Analysis; PCA, Principal Component Analysis.

a Specific disorder codes were added if this provides additional information.

**Table 8:** Cross-domain reviews.

**References**				**Units of analysis**							**PVS**				**NVS**						**Main aim of review^a^**	**Key findings**
	**Depressive disorders**	**Bipolar disorders**	**Anxiety disorders**	**Genes**	**Molecules**	**Cells**	**Circuits**	**Physiology**	**Behavior**	**Self-report**	**Domain level**	**Reward responsiveness**	**Reward learning**	**Reward valuation**	**Domain level**	**Acute threat**	**Potential threat**	**Sustained threat**	**Loss**	**Frustrative nonreward**		
Terbeck et al. (2015)	x		x		x						x				x						Elements: mGluR5 (metabotropic glutamate receptor 5). Aim: review of mGluR5 within RDoC (PVS, NVS, SP, and ARS)	Evidence for abnormal glutamate activity related to NVS and PVS: antagonistic mGluR5 intervention may have prominent anti-addictive, anti-depressive and anxiolytic effects; initial human clinical PET research: predisposition for psychiatric problems due to abnormal metabotropic glutamate activity
Langenecker et al. (2014)	x	x					x	x	x			x	x	x					x		Elements: PV circuitry (reward), NV circuitry (rumination). Aim: understanding the neurobiology of mood disorders	HC displayed preferential processing of positive stimuli (PV circuit), which resulted in a greater available range of approach behaviors and magnified regulatory capacities for appraisal, reappraisal, and the appropriate selection of alternative responses; MDD and BD: decreased preferential strengths of PV circuit and regulatory capacity, NV circuit and corresponding avoidance behaviors are more pronounced, PVS is over-/under-utilized in BD/MDD
Janiri et al. (2020) [meta-analysis]	x	x	x				x	x				x	x	x		x	x			x	Elements: task-related fMRI. Aim: neural phenotypes for highly comorbid mood and anxiety disorders; is their clinical overlap reflected at neurobiological level? Detection of transdiagnostic abnormalities in task-related brain activation	Dominant abnormality in mood, anxiety disorders, and PTSD: disruption in salience processing and inhibitory control; three transdiagnostic clusters of hypoactivation (inferior PFC/insula, inferior parietal lobule, putamen) → diagnostic differences only in putamen; three transdiagnostic clusters of hyperactivation (pgACC/dACC, left amygdala/PHC, left thalamus) → no diagnostic differences → RDoC domains/constructs did not contribute differently to any clusters
McTeague et al. (2020) [meta-analysis]	x	x	x				x				x									x	Elements: fMRI, PET, SPECT during emotional processing (230 reactivity, 56 emotional/cognitive, 18 regulation). Aim: a transdiagnostic quantitative meta-analysis of published neuroimaging data to investigate functional disruptions in the neural circuitry responsible for emotional processing across various tasks and psychiatric disorders	(1) Aberrant activation by disorder groupings: aberrant activation in the left amygdala and hippocampus in AD; BD associated with convergence in right amygdala and right vlPFC; MDD revealed no convergent patterns; (2) Hyper- vs. hypoactivation by disorder groupings: AD and MDD displayed overlapping hyperactivation in the left amygdala and the hippocampus;
																						(3) Neurocircuit disruption across major psychiatric disorders in key regions of adaptive emotional reactivity/regulation → these corresponded to “salience” network, ventral striatal/ventromedial prefrontal (reward) network and lateral orbitofrontal (nonreward) network

RDoC terms: ARS, Arousal and Regulatory Systems; NV, Negative Valence; NVS, Negative Valence Systems; PV, Positive Valence; PVS, Positive Valence Systems; RDoC, Research Domain Criteria; SP, Social Processes. Disorder: AD, Anxiety Disorder; BD, Bipolar Disorder; MDD, Major Depressive Disorder; PTSD, Post-Traumatic Stress Disorder. Molecules: mGluR5, Metabotropic Glutamate Receptor 5. Circuit: dACC, dorsal Anterior Cingulate Cortex; pgACC, pregenual Anterior Cingulate Cortex; PFC, Prefrontal Cortex; vlPFC, ventrolateral Prefrontal Cortex; PHC, Parahippocampal Cortex. Physiology: fMRI, functional Magnet Resonance Imaging; PET, Positron Emission Tomography; SPECT, Single Photon Emission Computed Tomography. Methods: HC, Healthy Controls.

a Specific disorder codes were added if this provides additional information.

### 3.3. Positive Valence Systems

We identified 17 publications (10 primary articles, seven review articles) addressing research on PVS in mood and anxiety disorders guided by the RDoC framework. All primary and two of the review articles included self-report measures (71%). The majority of these articles combined self-report with at least one additional unit of analysis, most commonly physiology or behavior. Only two publications included the cellular unit (12%, two reviews). In this scoping review, we did not identify any published primary research focusing on PVS-related genes or cells in mood and anxiety disorders that was oriented toward the RDoC framework. Review articles focusing on the RDoC framework and investigating PVS in mood and anxiety disorders mostly reviewed research investigating the circuit unit of analysis (35%, one primary article, five reviews). A total of 12 articles exclusively focused on patients suffering from depressive disorders or symptoms (71%; eight primary articles, four reviews). While none focused exclusively on patients with AD, five articles included more than one patient group (29%; two primary articles, three reviews).

Impaired hedonic experience as a marker of impaired reward responsiveness has proven to be a relevant PVS-related construct specifically in patients with MDD (Nakonezny et al., 2015; Barch et al., 2016; Nusslock and Alloy, 2017; Trøstheim et al., 2020). Hedonic experience has been confirmed to be a unidimensional factor (Nakonezny et al., 2015) that has been found to be a responsive target of exercise treatment (Toups et al., 2017). Literature suggests that depression-related hedonic impairments trigger deficits in other PVS mechanisms like anticipation, learning, effort, and action selection and that these impairments are associated with alterations in striatal dopamine and/or opioid signaling (Barch et al., 2016; Nusslock and Alloy, 2017). Orbitofrontal cortico-striatal circuits (OFC-striatal circuits) were found to be associated with reward valuation and dysfunctions were found in patients with MDD. Furthermore, this review found that abnormal activation in these circuits was modifiable through (non-)invasive brain stimulation techniques (Fettes et al., 2017). One study (Alexopoulos et al., 2015, 2016) focused on an RDoC-oriented neuroscience-driven psychotherapeutic intervention. In this study, reward-exposure served as an RDoC-based intervention in late life depression. This approach proved efficacious in eliciting changes in behavioral activation that in turn led to improvement of depressive symptoms during treatment and follow-up (Alexopoulos et al., 2015, 2016). Familial risk of depression and aberrant reward processing has been shown to have significant impact on individuals' reward processing which increases over the course of puberty (Nusslock and Alloy, 2017; Ethridge et al., 2021). Furthermore, aberrant reward sensitivity on a neural and behavioral level was associated with risk for depression (Baskin-Sommers and Foti, 2015). Furthermore, aberrant reward processing was linked to molecular alterations in the dopamine system and an increased vulnerability to late-life depression (Taylor et al., 2022). Depressive symptoms could be linked to increased reward valuation and reduced effort (drive) as well as reduced reward responsiveness (Nusslock et al., 2015; Nusslock and Alloy, 2017; Swope et al., 2020). However, regarding behavioral and circuit-related reward responsiveness, Langenecker et al. (2022) found no differences between patients with remitted mood disorders and healthy controls. According to the authors, these results therefore suggest that reward responsiveness may serve as a proximal marker for acute affective symptoms rather than being a trait or stable marker of patients with mood disorders. Regarding the RDoC construct reward valuation, the subconstructs motivation and energy - as constructs matchable to effort and drive - were suggested to be more clinically relevant compared to anhedonia/hedonic experience (Toups et al., 2017). Furthermore, decreased approach motivation (as part of reward valuation) could be related to unipolar depression, whereas increased approach motivation could be related to bipolar disorder with both mechanisms showing distinct neuronal correlates (Nusslock et al., 2015; Nusslock and Alloy, 2017). PVS functioning as measured by the newly developed Positive Valence Systems Scale (PVSS-21), was more strongly linked to symptoms of depression compared to symptoms of anxiety, was able to distinguish between depressed vs. non-depressed individuals, and predicted severity of anhedonia (Khazanov et al., 2020). Using an exploratory factor analysis (EFA) approach, several PVS factors were identified as significantly related to depressive symptoms (Olino et al., 2018). Notably, positive emotions showed the strongest negative association with depressive symptoms among all identified factors. High-frequency heart rate variability as a marker of disturbances in positive emotional functioning has been shown to exhibit greater intra-individual variation in patients with bipolar disorder compared to patients with MDD or healthy controls (Gruber et al., 2015).

### 3.4. Negative Valence Systems

We identified 17 publications (eight primary articles, nine reviews) investigating the role of NVS in mood and anxiety disorders within the RDoC framework. With one exception, all primary articles employed self-report measures (53%, seven primary articles, two reviews), regularly in conjunction with one or more additional units of analysis. We did not find any primary research examining MDD and AD on a molecular or cellular level, and only one paper that accounted for genetic influences by incorporating twin data. Reviews were mainly centered around physiological measures (71%, four primary articles, eight reviews) that were often combined with behavioral tasks (47%, three primary articles, five reviews), neuroimaging (41%, three primary articles, four reviews), or genomic aspects (29%, one primary article, four reviews). Only one review also integrated research on the cellular correlates of MDD. Four out of 17 articles exclusively focused on patients diagnosed with MDD (24%), four on patients with AD (24%), and eight articles included more than one group of patients (47%). There were no primary articles and only one review pertaining to NVS in BD patients (5%). All NVS subconstructs were studied across diagnostic categories and generally assessed using multiple units of analysis. Acute, potential, and sustained threat received the most empirical attention, with many review articles examining all of them together. Frustrative nonreward received the least empirical attention with only one primary article investigating the construct in relation to depressive symptoms.

For the NVS subconstruct acute threat, an fMRI study of patients with symptoms of depression and anxiety revealed transdiagnostic patterns of altered threat processing in the bilateral insula, the cingulate and the dorsolateral prefrontal cortex (MacNamara et al., 2017). Self-reported stress reactivity as a measure of potential threat was found to modulate risk for comorbid expressions of MDD and alcohol use disorders (AUD) via genetic and environmental factors (Ellingson et al., 2016). Sustained threat in the form of trauma and chronic stress was linked to alterations in protein expression, neurocircuitry, physiology, and behavior, with evidence suggesting specific modulations of amygdala activation and Hypothalamic–Pituitary–Adrenal axis (HPA-axis) reactivity, highlighting the role of this subconstruct in the development of mood as well as anxiety disorders (Ross et al., 2017; Sambuco et al., 2020). ERP studies showed that while individuals with internalizing psychopathologies exhibited certain transdiagnostic abnormalities in threat processing (Klumpp and Shankman, 2018), depressive and anxious disorders were marked by diagnosis-specific modulations in startle response (Vaidyanathan et al., 2012; Boecker and Pauli, 2019) that predicted the severity and the extent of psychopathology (Lang et al., 2016, 2018). Some of these abnormalities have been traced back to disorder-specific genetic alterations within the serotonergic system and the HPA-axis (Hamm et al., 2016). Two review articles regarding the NVS subconstruct loss discussed the role of rumination in MDD and BD patients and outlined potential approaches for the further study of depressive symptomatology within the RDoC framework (Silveira and Kauer-Sant'Anna, 2015; Woody and Gibb, 2015). As a behavioral component of loss, self-reported anhedonia was found to predict symptom severity for a broad range of psychiatric disorders, with particularly strong associations existing between anhedonia and depression (Guineau et al., 2022). A single article focused on Frustrative nonreward in conjunction with loss and potential threat, establishing these constructs as transdiagnostic features implicated in the development and change of depressive symptoms over the course of pregnancy and postpartum in a factor analysis of self-report questionnaire data (Cochran et al., 2020). Examining sustained threat and loss, a review of genetic influences on attentional bias showed that MDD and AD patients were characterized by disorder-specific alterations in attentional bias for affectively salient stimuli, and that the development of these differences was a consequence of environmental factors interacting with genes related to HPA-axis reactivity. Overall, research suggests the existence of transdiagnostic as well as disorder-specific dysfunctions in NVS domains across different units of analysis in MDD, AD, and BD patients.

### 3.5. Cross-domain Positive and Negative Valence Systems

We identified nine publications (five primary articles, four review articles) covering cross-domain research on PVS and NVS in mood and anxiety disorders in accordance with the RDoC framework. All primary articles but none of the reviews reported results from self-report questionnaires or interviews (50%). Behavioral measures also mainly played a role in primary research (40%, three primary articles, one review) while review articles again put more emphasis on circuitry (40%, one primary article, three review articles) and physiology (30%, one primary article, two reviews). Two articles also reported findings on the molecular level (20%, one primary article, one review). We did not identify relevant cross-domain research regarding genetic and cellular correlates of PVS and NVS functioning. Cross-domain articles reported mainly on samples including more than one patient group (89%; four primary articles, four reviews). One primary article exclusively focused on patients suffering from depressive disorders (11%). None of the identified articles focused exclusively on patients with AD. Finally, two out of nine articles could not be allocated to a specific RDoC (sub-)construct. Hence, we reported the results on the domain level. All other cross-domain articles focused on the broad spectrum of PVS- and NVS (sub-) constructs.

With regards to factorial analytic studies, one article investigating the multimodal factorial structure underlying a broad test battery comprised of self-report, behavioral, and neuroimaging assessments to capture PVS and NVS functioning in patients with mood and anxiety disorder symptoms failed to identify a cross-modal latent structure and attributed this to challenges in the RDoC approach (Peng et al., 2021). However, Paulus et al. (2017) reported finding two independent “meta”-dimensions of PVS and NVS using a factorial analytic approach on self-report and behavioral data. Likewise focusing on self-report and behavioral units. Förstner et al. (2022) found a structure of four latent and transnosological factors (PVS, NVS, CS, and SP) using a confirmatory factor analysis (CFA) approach, although these factors were not cross-modal. Differential associations between PV and NV symptom scores and clinical impairment, antidepressant response, and inflammation-related immunomarkers were revealed in a sample of MDD patients by Medeiros et al. (2020). Specifically, PV symptoms were linked to higher cognitive and physical impairment, showed associations to a greater number of inflammatory markers, and were more responsive to treatment with antidepressants, while NV symptoms were linked to younger age and a higher rate of comorbid anxiety symptoms. Wenzel et al. (2022) investigated self-reported PVS and NVS functioning in perinatal women: Trait- and state-like NVS functioning (potential threat) as well as state-like PVS functioning (reward valuation) were linked to worse depressive symptoms, while trait- and state-like NVS functioning (potential threat) were also linked to higher anxiety scores. Therefore, Wenzel et al. (2022) suggested potential threat as a transdiagnostic feature of perinatal anxiety and depression, whereas reward valuation was suggested to be a disease- or symptom-specific feature of perinatal depression. Compared to healthy controls, individuals with MDD or BD showed no preferential processing of positive stimuli (PVS circuit), while NVS circuitry was more pronounced (Langenecker et al., 2014). PVS mechanisms may be more often investigated in BD and underutilized in MDD (Langenecker et al., 2014). In a meta-analysis of 226 fMRI studies, Janiri et al. (2020) identified transdiagnostic neural phenotypes that are characteristic of patients with mood, anxiety, and posttraumatic stress disorders: In particular, the authors describe clusters of hypoactivation in the inferior prefrontal cortex, the inferior parietal lobule, and the putamen as well as clusters of hyperactivation in the left amygdala/parahippocampal gyrus, the left thalamus, and the dorsal anterior cingulate cortex, supporting the hypothesis of transdiagnostic neuronal disease mechanisms. However, RDoC domains did not contribute differentially to these clusters, which points to the clusters being domain-independent. Regarding PVS- and NVS-circuits, transdiagnostic patterns of disrupted activity were identified in the ventrolateral, ventromedial and dorsomedial prefrontal cortex, the amygdala, and thalamo-cortical networks by McTeague et al. (2020) for MDD and AD, while they also found evidence for disease-specific aberrant activation for AD, BD, and MDD. One review investigating the molecular basis of PVS and NVS in mood and anxiety disorders identified abnormal glutamate activity related to PVS and NVS (Terbeck et al., 2015).

## 4. Discussion

### 4.1. Summary of evidence

Our scoping review aimed to explore the recent research activity on the RDoC PVS and NVS in mood and anxiety disorders. We identified 43 publications that investigated positive and negative valence in mood and anxiety disorders, utilizing various measures from a range of genetic, molecular, neuronal, physiological, behavioral, and self-report approaches. Primary articles chiefly employed self-report questionnaires and interviews, often in conjunction with behavioral data. Reviews frequently included results from the molecules, circuitry, and physiology units of analysis. The structural and functional imaging literature included in this review highlighted the essential role of specific cortical frontal brain structures and of certain subcortical limbic structures, such as the amygdala and the hippocampus, in impaired emotional processing among patients with mood and anxiety disorders. Both reward- and threat-related processing were investigated in terms of genetic and molecular aspects, underlying circuitry, physiological responses, observed behavior, and self-reported symptoms, with many articles examining relationships between multiple units of analysis. Transdiagnostic as well as diagnosis-specific anomalies could be demonstrated -inter alia- on the levels of protein expression, concentration of hormones and immunomarkers, neural activity in brain areas associated with salience and reward, HPA-axis activation, behavioral indicators like attentional bias and startle response, and self-reported reward and threat sensitivity. However, identifying cross-modal constructs to characterize PVS and NVS functioning has proven challenging, an issue that is exemplified by a number of factor analytic studies reporting a lack of coherence in latent structure between tasks and measurement levels. We identified one intervention study testing the effectiveness of Engage therapy, an approach to improve symptoms connected to the positive valence domain by targeting the neural mechanisms underlying disordered emotional processing in late-life depression.

Both depressive and anxiety disorders were actively studied in the context of NVS, with a particular emphasis on the subconstructs of acute, potential, and sustained threat for anxious symptoms and loss for depressive symptoms. As we only identified one review investigating NVS in bipolar disorders, our scoping review highlights the lack of primary articles on NVS research focused on BD. Additionally, the review underscores the scarcity of research on frustrative nonreward in general, a gap initially identified by Carcone and Ruocco (2017). However, these research gaps could also be due to limitations in the search strategy employed, i.e., not explicitly searching for frustrative nonreward. There was limited research on the psychopathology of anxiety disorders within the PVS. Findings were largely connected to anxiety-related NVS constructs that cut across different AD, revealing a distinction between fear- or phobic-based disorders from non-phobic anxiety disorders. While mood disorders were widely studied in regards to both PVS and NVS, the number of publications exploring the psychopathology of BD was considerably smaller than those for MDD.

In relation to the Tripartite Model of Anxiety and Depression proposed by Clark and Watson (1991), we focus on reporting three selected key findings. Observed diagnosis-specific modulations in startle responses, as a marker of acute threat (NVS), enable to distinguish between depressive and anxiety disorders (Vaidyanathan et al., 2012; Boecker and Pauli, 2019), while this subconstruct also showed transdiagnostic patterns in an fMRI setting (MacNamara et al., 2017). These results show that RDoC subconstructs such as acute threat can be an additional markers distinctive of AD, as is physiological hyperarousal in the Tripartite Model. Impaired hedonic experience, indicative of impaired reward responsiveness (PVS), has been identified as a useful marker (Nakonezny et al., 2015; Barch et al., 2016; Nusslock and Alloy, 2017; Trøstheim et al., 2020) for distinguishing depressive symptoms from other mood and anxiety disorder symptoms, which is consistent with Tripartite Model assumptions. The relationship between reward processing and depressive symptoms in the context of PVS (Nusslock et al., 2015; Nusslock and Alloy, 2017; Swope et al., 2020) may be complex and requires further investigation in future research, as reward responsiveness may serve as a more proximal marker for acute affective symptoms such as anhedonia rather than being a stable trait marker of patients with mood disorders (Langenecker et al., 2022).

The findings in our review align with the work of Carcone and Ruocco (2017), who stated that RDoC-related publications typically investigate a single construct using multiple units of analysis, examine relationships between constructs and/or elements, or apply a transdiagnostic approach to measure them. More recent reviews of RDoC research tend to adopt a diagnosis-focused approach. For instance, Wei and Roodenrys (2021) focused on anxiety-related research, and Tschida and Yerys (2021) pursued a combined domain and diagnosis approach to explore PVS in autism spectrum disorder. In comparison, in our review we pursued a more transnosological approach. The number of articles originating from outside the US has increased. This increasing number originating from outside the US suggests that the scientific community is increasingly embracing the dimensional research approach by integrating it into their research efforts in recent years (Carcone and Ruocco, 2017).

### 4.2. Limitations

This scoping review has some limitations. This review was not intended to provide a comprehensive overview of all research on PVS and NVS in mood and anxiety disorders. Rather, our search strategy was focused specifically on PVS and NVS domain level. Therefore, we did not conduct keyword searches for specific PVS and NVS constructs or subconstructs, which may have resulted in the exclusion of relevant publications. Another limitation lies in the exclusion of studies that did not provide a clear assignment of their research to specific RDoC constructs (e.g., BIS/BAS). This exclusion criterion is not completely objectifiable and therefore the current review we may have excluded single RDoC-related results. Of note, the present review included RDoC related articles that specifically focused on PVS and NVS on the domain level. The aim of this review was not to synthesize the entire literature on symptoms or constructs related to transdiagnostic or RDoC associated domains. Specifically, we focused on articles explicitly investigating PVS and NVS domains but did not search for all articles that for example investigated fear in mood and anxiety disorders. Therefore, the present review does not cover all transdiagnostic issues related to mood and anxiety disorder symptoms (e.g., Sindermann et al., 2021; van Tol et al., 2021), but reflects research from the last decade conceptualizing PVS and NVS on the domain level only. Given our primary aim was to conduct a scoping review of articles with a direct mention of the RDoC approach, we did not systematically include primary articles included in reviews or meta-analyses presented here, but extracted relevant RDoC information from these articles: Be it in relation to individual articles within the listed reviews/meta-analyses or be it of results or conclusions of the authors of these reviews/meta-analyses. While this approach may have hampered the generalizability and precision of our findings with respect to all available data, we would argue that we could therefore better present the current state of research with regard to RDoC research efforts, which is also in line with our main aim, with value-added information from reviews/meta-analyses that are themselves RDoC-related but whose primary articles would not have been selected based on our eligibility criteria. A further limitation arises from the NIMH's update of the PVS domain. We mapped articles from before 2017 to the revised PVS structure released by the NIMH in 2017. As a result, it is possible that some articles may have been mapped to the PVS domain differently than originally intended by the authors. An additional limitation may arise from the incorporation of heterogenous research studies. However, given the nature of this review we could not address heterogeneity on a quantitative level. Lastly, as no systematic review was conducted, there was no quality assessment of the included studies, which is an inherent limitation of a scoping review.

## 5. Conclusions

The RDoC initiative was proposed as a translational framework for psychopathology research with the goal of addressing issues related to symptom-based diagnostic categories by shifting emphasis to dimensions of human functioning defined by observable behavior as well as neurobiological indicators (Cuthbert, 2020). This scoping review shows that overall, mood and anxiety disorders are actively studied in the context of PVS and NVS within the RDoC framework. In line with the integrative approach of the RDoC initiative, psychological constructs related to mood and anxiety disorders were typically examined across different units of analysis, with the majority of publications incorporating two or more measures to capture multiple facets of dysregulated functioning. While molecular, genetic, and physiological aspects were mainly investigated via review articles analyzing large bodies of research through the lens of the RDoC framework, current primary articles focused on self-report, behavioral, and—to a lesser extent—physiological measures as well.

It is clear that the RDoC initiative is influencing the direction of research into diagnosis-specific as well as transdiagnostic features of mood and anxiety disorders. This trend is particularly evident in the US, although our review suggests that the RDoC framework is increasingly being adopted in other countries as well. A major challenge for future research will be the translation of RDoC-guided findings into clinical practice (Pacheco et al., 2022). In this regard, the present scoping review emphasizes the potential of further research into depressive and anxious symptomatology conducted within the RDoC framework to potentially catalyze the development of neuroscience-driven interventions that target PVS and NVS functioning in alignment with current advancements in precision medicine approaches to diagnosis, treatment, and prevention of mood and anxiety disorders.

## Data availability statement

The original contributions presented in the study are included in the article/Supplementary material, further inquiries can be directed to the corresponding author.

## Author contributions

SB, BF, MR, and MT contributed to the conception and design of the study. MT, MR, and KK-S mentored the research project. SB prepared the review, ran the database searches, organized the studies, created the tables and figure, and wrote the first draft of the manuscript. SB, BF, LS, and MT performed all steps of the review: screening, eligibility, data extraction, and synthesis of search results into the tables. All authors substantially contributed to manuscript revision, read, and approved the submitted version.
