# Supplementary material for: Mood and anxiety disorders within the Research Domain Criteria framework of Positive and Negative Valence Systems: a scoping review
Source: Front Hum Neurosci. 2023 Jun 2;17:1184978. doi: 10.3389/fnhum.2023.1184978 (PMC10272468; doi:10.3389/fnhum.2023.1184978)
Supplement: Supplementary file 2 [file Data_Sheet_2.pdf]

Supplementary Material B

Mood and anxiety disorders within the Research Domain Criteria framework of Positive and Negative Valence Systems: a scoping review

Sarah Jane Böttger*, Bernd R. Förstner, Laura Szalek, Kristin Koller-Schlaud, Michael A. Rapp and Mira Tschorn

*** Correspondence:** Sarah Jane Böttger: sboettger@uni-potsdam.de

1 Scoping review protocol

Scoping review details	
Scoping review title:	Mood and Anxiety Disorders within the Research Domain Criteria framework of Positive and Negative Valence Systems: a scoping review
Scoping review objectives:	Scoping literature review of mood and anxiety disorders in relation to positive and negative valence
Scoping review questions:	What is the state of published research investigating the role of PVS and NVS in MAD using the RDoC framework?
**Inclusion/Exclusion criteria**	
Population:	Individuals with symptoms of mood (depression, bipolar) or anxiety (anxiety, phobia) disorders; adults (18 years and older)
Concept:	Outcome measures of positive valence or positive affect, and negative valence or negative affect, with reference to RDoC; all units of analysis (genes, molecules, cells, circuits, physiology, behavior, self-report)
Context:	Open
Type of evidence source:	Empirical research; peer-reviewed publications
**Covidence: Title and abstract screening**	
Keywords for inclusion [a]:	depression, bipolar, anxiety, phobia, phobic, panic, human, adults, positive valence, negative valence, positive valence systems, negative valence systems, RDoC, research domain criteria, threat, fear, loss, nonreward, anhedonia, trauma, guilt, crying, rumination, sadness, withdrawal, shame, worry, morbid thoughts, aggression, reward, habit, drive, self-report, schedule, questionnaire, interview, scale, molecule, cell, circuit, physiology

Keywords for exclusion:	animal, rats, mice, rodents, children, infants, youth, pediatric schizophrenia, borderline, PTSD, OCD, autism
Study tags for inclusion:	RDoC, genetic measures, molecule measures, neuronal measures (circuit measures), behavior measures, self-report measures, paradigms, positive valence, negative valence, depression, bipolar, anxiety, phobia
Covidence®: Full-text screening	
Exclusion reasons:	wrong population (other disorder), wrong population (healthy participants), wrong population (age), wrong population (animal studies), wrong domain(s) assessed (irrelevant research), irrelevant to research objectives (irrelevant research), no empirical research, research not found (no full text available)
Data charting process	
Evidence source details and characteristics:	Author/s, publication year, country, language, research design, disorder, aim, (outcome) measures, RDoC domain and (sub-)constructs, findings relevant to the research objectives
Details extracted from source of evidence:	Disorder, domain, constructs, subconstructs, units of analysis, elements, findings

The Scoping review protocol was developed using the Joanna Briggs Institute recommendations (Peters et al., 2020). NVS = Negative Valence Systems; PVS = Positive Valence Systems; RDoC = Research Domain Criteria; OCD = Obsessive-compulsive disorder; PTSD = Post-Traumatic Stress Disorder.

[a] A selection of keywords of the Positive Valence Systems and Negative Valence Systems domains, constructs, subconstructs and elements of different units of analysis were handpicked in accordance to the RDoC matrix (NIMH, 2023).

2 REFERENCES

NIMH (2023). *RDoC Matrix*. Accessed March 10, 2023, https://www.nimh.nih.gov/research/research-funded-by-nimh/rdoc/constructs/rdoc-matrix.shtml

Peters, M., Godfrey, C., McInerney, P., Munn, Z., Trico, A., and Khalil, H. (2020). “Chapter 11: Scoping Reviews,” in *JBI Manual for Evidence Synthesis*, eds. E. Aromataris, and Z. Munn (JBI).
