# Supplementary material for: Mood and anxiety disorders within the Research Domain Criteria framework of Positive and Negative Valence Systems: a scoping review
Source: Front Hum Neurosci. 2023 Jun 2;17:1184978. doi: 10.3389/fnhum.2023.1184978 (PMC10272468; doi:10.3389/fnhum.2023.1184978)
Supplement: Supplementary file 3 [file Data_Sheet_3.pdf]

Supplementary Material C

Mood and anxiety disorders within the Research Domain Criteria framework of Positive and Negative Valence Systems: a scoping review

Sarah Jane Böttger[*], Bernd R. Förstner, Laura Szalek, Kristin Koller-Schlaud, Michael A. Rapp and Mira Tschorn

*** Correspondence:** Sarah Jane Böttger: sboettger@uni-potsdam.de

1 Search Strategy

A detailed search strategy for all sources searched is presented in the following tables 1-3.

Table 1: Search Strategy used (via EBSCOhost) in PsychInfo, PsychArticles and PSYNDEX

Search component	Search terms	Search results on April 26, 2021 (*N*)			Search results on January 21, 2023 (*N* (additional *n*))		
		PsychInfo	PsychArticles	PSYNDEX	PsychInfo	PsychArticles	PSYNDEX
**Search 1**							
S1	AB ("depression" or "depressive disorder*" or "depressive symptom*" or "major depressive disorder") OR AB "affective disorder*" OR AB "mood disorder*" OR AB ("bipolar disorder*" or "bipolar" i or "bipolar ii" or "manic depression" or "bipolar affective disorder*" or "bipolar depression") OR AB ("mania" or "manic" or "manic episode") OR AB ("anxiety disorder*" or "anxiety") OR AB ("phobia" or "phobic disorder*") OR AB ("panic disorder*")	428,433	16,065	21,023	466,901	17,461	23,517
S2	AB "rdoc" OR AB "research domain criteria"	695	40	32	829	53	51
S3	AB "positive valence" OR AB "negative valence"	1,528	126	173	1,736	148	210
S4	S1 AND S2 AND S3	49	2	2	59 (10)	3 (1)	3 (1)
**Search 2**							
S5	AB "valence" OR AB "affect*" OR AB "emotion*"	734,770	34,792	42,982	806,829	37,630	48,052
S6	S1 AND S2 AND S5	111	7	4	133 (22)	8 (1)	7 (3)
**Conjunction of Search 1 and 2**							
S7	S4 OR S6	111	7	4	133 (22)	8 (1)	7 (3)

The search was conducted on April 26, 2021 and updated on January 21, 2023. AB = Abstract; rdoc = Research Domain Criteria.

Table 2: Search Strategy used in PubMed

Search component	Search terms	Search results on April 26, 2021 (*N*)	Search results on January 21, 2023 (*N* (additional *n*))
**Search 1**			
#1	("depression"[Title/Abstract]) OR ("depressive disorder*"[Title/Abstract]) OR ("affective disorder*"[Title/Abstract]) OR ("mood disorder*"[Title/Abstract]) OR ("bipolar"[Title/Abstract]) OR ("bipolar disorder*"[Title/Abstract]) OR ("maniac"[Title/Abstract]) OR ("manic"[Title/Abstract]) OR ("anxiety"[Title/Abstract]) OR ("anxiety disorder*"[Title/Abstract]) OR ("phobia*"[Title/Abstract]) OR ("phobic disorder*"[Title/Abstract]) OR ("panic disorder*"[Title/Abstract])	558,992	635,001
#2	("rdoc"[Title/Abstract]) OR ("research domain criteria"[Title/Abstract])	855	1,061
#3	("positive valence"[Title/Abstract]) OR ("negative valence"[Title/Abstract])	1,238	1,538
#4	#1 AND #2 AND #3	64	86 (22)
**Search 2**			
#5	("valence"[Title/Abstract]) OR ("affect*"[Title/Abstract]) OR ("emotion*"[Title/Abstract])	2,150,390	2,451,343
#6	#1 AND #2 AND #5	147	191 (44)
**Conjunction of Search 1 and 2**			
#7	#4 OR #6	147	191 (44)

The search was conducted on April 26, 2021 and updated on January 21, 2023. AB = Abstract; rdoc = Research Domain Criteria.

Table 3: Search Strategy used in Web of Science

Search component	Search terms	Search results on April 26, 2021 (*N*)	Search results on January 21, 2023 (*N* (additional *n*))
**Search 1**			
#1	(AB="depression") OR (AB="depressive disorder*") OR (AB="bipolar") OR (AB="affective disorder*") OR (AB="mood disorder*") OR (AB="anxiety") OR (AB="phobia") OR (AB="phobic disorder*") OR (AB="panic disorder*")	505,695	585,665
#2	(AB="rdoc") OR (AB="research domain criteria")	649	808
#3	(AB="positive valence") OR (AB="negative valence")	1,577	1,951
#4	#1 AND #2 AND #3	50	68 (18)
**Search 2**			
#5	(AB="valence") OR (AB="affect*") OR (AB="emotion*")	2,929,932	3,399,188
#6	#1 AND #2 AND #5	105	135 (30)
**Conjunction of Search 1 and 2**			
#7	#4 OR #6	105	135 (30)

The search was conducted on April 26, 2021 and updated on January 21, 2023. AB = Abstract; rdoc = Research Domain Criteria.
