# Supplementary material for: Mood and anxiety disorders within the Research Domain Criteria framework of Positive and Negative Valence Systems: a scoping review
Source: Front Hum Neurosci. 2023 Jun 2;17:1184978. doi: 10.3389/fnhum.2023.1184978 (PMC10272468; doi:10.3389/fnhum.2023.1184978)
Supplement: Supplementary file 4 [file Data_Sheet_4.pdf]

Supplementary Material D

Mood and anxiety disorders within the Research Domain Criteria framework of Positive and Negative Valence Systems: a scoping review

Sarah Jane Böttger*, Bernd R. Förstner, Laura Szalek, Kristin Koller-Schlaud, Michael A. Rapp and Mira Tschorn

*** Correspondence:** Sarah Jane Böttger: sboettger@uni-potsdam.de

1 Sources excluded following title and abstract screening

Al Zoubi, O., Ki Wong, C., Kuplicki, R. T., Yeh, H.-W., Mayeli, A., Refai, H., et al. (2018). Predicting Age From Brain EEG Signals-A Machine Learning Approach. *Front Aging Neurosci* 10, 184. doi: 10.3389/fnagi.2018.00184

Al-Khalil, K., Vakamudi, K., Witkiewitz, K., and Claus, E. D. (2021). Neural Correlates of Alcohol Use Disorder Severity among Non-Treatment Seeking Heavy Drinkers: An Examination of the Incentive Salience and Negative Emotionality Domains of the Alcohol and Addiction Research Domain Criteria. *Alcohol Clin Exp Res*. doi: 10.1111/acer.14614

Altimir, C., La Cerda, C. de, and Dagnino, P. (2021). "The functional domain of affect regulation," in *Depression and personality dysfunction: An integrative functional domains perspective*, eds. G. de La Parra, P. Dagnino, and A. Behn (Cham: Springer Nature Switzerland AG), 33–69.

Alvarez-Fernandez, S., Brown, H. R., Zhao, Y., Raithel, J. A., Bishop, S. L., Kern, S. B., et al. (2017). Perceived social support in adults with autism spectrum disorder and attention-deficit/hyperactivity disorder. *Autism Res* 10, 866–877. doi: 10.1002/aur.1735

Bailey, J., and Baker, S. T. (2020). A synthesis of the quantitative literature on autistic pupils' experience of barriers to inclusion in mainstream schools. *JOURNAL OF RESEARCH IN SPECIAL EDUCATIONAL NEEDS* 20, 291–307. doi: 10.1111/1471-3802.12490

Barch, D. M., Pagliaccio, D., and Luking, K. (2019). "Positive valence system dysregulation in psychosis: A comparative analysis," in *The Oxford handbook of positive emotion and psychopathology*, ed. J. Gruber (New York, NY: Oxford University Press), 253–283.

Barch, D. M., Pagliaccio, D., and Luking, K. R. (2018). "Motivational impairments in psychotic and depressive pathology: Psychological and neural mechanisms," in *Neurobiology of abnormal emotion and motivated behaviors: Integrating animal and human research*, eds. S. Sangha, and D. Foti (San Diego, CA: Elsevier Academic Press), 279–304.

Beaudreau, S. A., Hantke, N. C., Mashal, N., Gould, C. E., Henderson, V. W., and O'Hara, R. (2017). Unlocking Neurocognitive Substrates of Late-Life Affective Symptoms Using the Research Domain Criteria: Worry Is an Essential Dimension. *Front Aging Neurosci* 9, 380. doi: 10.3389/fnagi.2017.00380

Bebko, G., Bertocci, M., Chase, H., Dwojak, A., Bonar, L., Almeida, J., et al. (2015). Decreased amygdala-insula resting state connectivity in behaviorally and emotionally dysregulated youth. *Psychiatry Res* 231, 77–86. doi: 10.1016/j.pscychresns.2014.10.015

Bergsholm, P. (2016). Is schizophrenia disappearing? The rise and fall of the diagnosis of functional psychoses: an essay. *BMC Psychiatry* 16, 387. doi: 10.1186/s12888-016-1101-5

Bernard, J. A., and Mittal, V. A. (2015). Updating the research domain criteria: the utility of a motor dimension. *Psychol Med* 45, 2685–2689. doi: 10.1017/S0033291715000872

Biele, G., Overgaard, K. R., Friis, S., Zeiner, P., and Aase, H. (2022). Cognitive, emotional, and social functioning of preschoolers with attention deficit hyperactivity problems. *BMC Psychiatry* 22, 78. doi: 10.1186/s12888-021-03638-9

Blom, E. H., Duncan, L. G., Ho, T. C., Connolly, C. G., LeWinn, K. Z., Chesney, M., et al. (2014). The development of an RDoC-based treatment program for adolescent depression: 'Training for Awareness, Resilience, and Action' (TARA). *Front Hum Neurosci* 8. doi: 10.1037/t55280-000.

Blom, E. H., Tymofiyeva, O., Chesney, M. A., Ho, T. C., Moran, P., Connolly, C. G., et al. (2017). Feasibility and preliminary efficacy of a novel RDoC-based treatment program for adolescent depression: 'Training for Awareness Resilience and Action' (TARA)—A pilot study. *Front Psychiatry* 7. doi: 10.1037/t02080-000;

Bluett, R. J. *The Endocannabinoid, 2-arachidonoylglycerol, Regulates Resilience to Stress-induced Anxiety.* ProQuest Information & Learning.

Brotman, M. A., Sharif-Askary, B., Dickstein, D. P., and Leibenluft, E. (2016). “Biological factors In bipolar disorder in childhood and adolescence,” in *Bipolar disorders: Basic mechanisms and therapeutic implications., 3rd ed*, eds. J. C. Soares, and A. H. Young (New York, NY: Cambridge University Press), 219–233.

Brückl, T. M., Spoormaker, V. I., Sämann, P. G., Brem, A.-K., Henco, L., Czamara, D., et al. (2020). The biological classification of mental disorders (BeCOME) study: a protocol for an observational deep-phenotyping study for the identification of biological subtypes. *BMC Psychiatry* 20, 213. doi: 10.1186/s12888-020-02541-z

Budde, M., Anderson-Schmidt, H., Gade, K., Reich-Erkelenz, D., Adorjan, K., Kalman, J. L., et al. (2019). A longitudinal approach to biological psychiatric research: The PsyCourse study. *Am J Med Genet B Neuropsychiatr Genet* 180, 89–102. doi: 10.1002/ajmg.b.32639

Bufferd, S. J., Dyson, M. W., Hernandez, I. G., and Wakschlag, L. S. (2016). “Explicating the 'developmental' in preschool psychopathology,” in *Developmental psychopathology: Maladaptation and psychopathology., Vol. 3, 3rd ed*, ed. D. Cicchetti (Hoboken, NJ: John Wiley & Sons, Inc), 152–186.

Capuron, L., and Castanon, N. (2017). Role of Inflammation in the Development of Neuropsychiatric Symptom Domains: Evidence and Mechanisms. *Curr Top Behav Neurosci* 31, 31–44. doi: 10.1007/7854_2016_14

Carvalho, A. F., Dimellis, D., Gonda, X., Vieta, E., Mclntyre, R. S., and Fountoulakis, K. N. (2014). Rapid cycling in bipolar disorder: a systematic review. *J Clin Psychiatry* 75, e578-86. doi: 10.4088/JCP.13r08905

Clinton, S. M., Shupe, E. A., Glover, M. E., Unroe, K. A., McCoy, C. R., Cohen, J. L., et al. (2021). Modeling heritability of temperamental differences, stress reactivity, and risk for anxiety and depression: Relevance to research domain criteria (RDoC). *Eur J Neurosci*. doi: 10.1111/ejn.15158

Cohen, A. S., Najolia, G. M., Kim, Y., and Dinzeo, T. J. (2012). On the boundaries of blunt affect/alogia across severe mental illness: implications for Research Domain Criteria. *Schizophr Res* 140, 41–45. doi: 10.1016/j.schres.2012.07.001

Cope, Z. A., Powell, S. B., and Young, J. W. (2016). Modeling neurodevelopmental cognitive deficits in tasks with cross-species translational validity. *Genes Brain Behav* 15, 27–44. doi: 10.1111/gbb.12268

Craighead, W. E. (2013). Editor's final comments. *Clin Psychol (New York)* 20, 359–360. doi: 10.1111/cpsp.12046

Crocq, M.-A. (2017). The history of generalized anxiety disorder as a diagnostic category. *Dialogues Clin Neurosci* 19, 107–116. doi: 10.31887/DCNS.2017.19.2/macrocq

Da Silva, S., Apatsidou, A., Saperia, S., Siddiqui, I., Jeffay, E., Voineskos, A. N., et al. (2018). An Examination of the Multi-Faceted Motivation System in Healthy Young Adults. *Front Psychiatry* 9, 191. doi: 10.3389/fpsyt.2018.00191

Di Cerbo, A. (2021). Letter to the Editor: CONVERGENCES AND DIVERGENCES IN THE ICD-11 VS. DSM-5 CLASSIFICATION OF MOOD DISORDERS. *Turk Psikiyatri Derg* 32, 293–295. doi: 10.5080/u26899

Dieterich, A. *Chronic stress exposure impairs reward and motivation behaviors in mice.* ProQuest Information & Learning.

Doom, J. R., Rozenman, M., Fox, K. R., Phu, T., Subar, A. R., Seok, D., et al. (2021). The Transdiagnostic Origins of Anxiety and Depression During the Pediatric Period: Linking NIMH Research Domain Criteria (RDoC) Constructs to Ecological Systems. *Dev Psychopathol* 33, 1599–1619. doi: 10.1017/s0954579421000559

Draps, M., Sescousse, G., Wilk, M., Obarska, K., Szumska, I., Żukrowska, W., et al. (2021). An empirical study of affective and cognitive functions in Compulsive Sexual Behavior Disorder. *J Behav Addict* 10, 657–674. doi: 10.1556/2006.2021.00056

Dwyer, K. R. *White matter connectivity and social cognitive impairment in a transdiagnostic sample.* ProQuest Information & Learning.

Edelstyn, N., Di Rosa, E., and Martini, A. (2023). Identifying the Basic Dimensions of Medication-Triggered Impulsive Compulsive Behaviours in Parkinson's Disease. *Eur Neurol*, 1–4. doi: 10.1159/000528900

Ekbäck, E., Granåsen, G., Svärling, R., Blomqvist, I., and Henje, E. (2021). Clinical Effectiveness of Training for Awareness Resilience and Action Online Compared to Standard Treatment for Adolescents and Young Adults With Depression: Study Protocol and Analysis Plan for a Pragmatic, Multi-Center Randomized Controlled Superiority Trial. *Front Psychiatry* 12, 674583. doi: 10.3389/fpsyt.2021.674583

Ekhtiari, H., Kuplicki, R., Yeh, H.-W., and Paulus, M. P. (2019). Physical characteristics not psychological state or trait characteristics predict motion during resting state fMRI. *Sci Rep* 9, 419. doi: 10.1038/s41598-018-36699-0

Fanselow, M. S. (2022). Negative valence systems: sustained threat and the predatory imminence continuum. *Emerg Top Life Sci* 6, 467–477. doi: 10.1042/ETLS20220003

Fawcett, J. (2011). Nature, nurture, and treatment specificity. *Psychiatr Ann* 41, 517. doi: 10.3928/00485713-20111017-01

Feld, G. B., and Feige, B. (2021). Arousal and regulatory systems in the system of research domain criteria. *Nervenarzt* 92, 907–914. doi: 10.1007/s00115-021-01160-7

Fernandez, K. C., Morrison, A. S., and Gross, J. J. (2019). “Emotion regulation,” in *The Cambridge handbook of anxiety and related disorders*, ed. B. O. Olatunji (New York, NY: Cambridge University Press), 282–305.

Fung, L. K., and Reiss, A. L. (2016). Moving Toward Integrative, Multidimensional Research in Modern Psychiatry: Lessons Learned From Fragile X Syndrome. *Biol Psychiatry* 80, 100–111. doi: 10.1016/j.biopsych.2015.12.015

Gandy, K., Kim, S., Sharp, C., Dindo, L., Maletic-Savatic, M., and Calarge, C. (2017). Pattern Separation: A Potential Marker of Impaired Hippocampal Adult Neurogenesis in Major Depressive Disorder. *Front Neurosci* 11, 571. doi: 10.3389/fnins.2017.00571

Gao, M. M., Ostlund, B., Brown, M. A., Kaliush, P. R., Terrell, S., Vlisides-Henry, R. D., et al. (2021). Prenatal maternal transdiagnostic, RDoC-informed predictors of newborn neurobehavior: Differences by sex. *Dev Psychopathol*, 1–12. doi: 10.1017/S0954579420002266

Geller, W. N. *Resting-state functional connectivity in anhedonic depression.* ProQuest Information & Learning.

Giménez-Llort, L., Santana-Santana, M., and Bayascas, J. R. (2020). The Impact of the PI3K/Akt Signaling Pathway in Anxiety and Working Memory in Young and Middle-Aged PDK1 K465E Knock-In Mice. *Front Behav Neurosci* 14, 61. doi: 10.3389/fnbeh.2020.00061

Girgis, F., Lee, D. J., Goodarzi, A., and Ditterich, J. (2018). Toward a Neuroscience of Adult Cognitive Developmental Theory. *Front Neurosci* 12, 4. doi: 10.3389/fnins.2018.00004

Glannon, W. (2015). Neuromodulation and the mind-brain relation. *Front Integr Neurosci* 9.

Goodwin, G. M., Holmes, E. A., Andersson, E., Browning, M., Jones, A., Lass-Hennemann, J., et al. (2018). From neuroscience to evidence based psychological treatments - The promise and the challenge, ECNP March 2016, Nice, France. *Eur Neuropsychopharmacol* 28, 317–333. doi: 10.1016/j.euroneuro.2017.10.036

Grant, B. R., O'Loughlin, K., Holbrook, H. M., Althoff, R. R., Kearney, C., Perepletchikova, F., et al. (2020). A multi-method and multi-informant approach to assessing post-traumatic stress disorder (PTSD) in children. *Int Rev Psychiatry* 32, 212–220. doi: 10.1080/09540261.2019.1697212

Gur, R. E., Moore, T. M., Calkins, M. E., Ruparel, K., and Gur, R. C. (2017). Face Processing Measures of Social Cognition: A Dimensional Approach to Developmental Psychopathology. *Biol Psychiatry Cogn Neurosci Neuroimaging* 2, 502–509. doi: 10.1016/j.bpsc.2017.03.010

Hasratian, A. M., Nordberg, H. O., Meuret, A. E., and Ritz, T. (2021). Fear and Coping in Students during the Early Stages of the COVID-19 Pandemic: A Combined Cross-Sectional and Longitudinal Study. *Int J Environ Res Public Health* 18. doi: 10.3390/ijerph18126551

Hegerl, U., and Hensch, T. (2017). Why do stimulants not work in typical depression? *Aust N Z J Psychiatry* 51, 20–22. doi: 10.1177/0004867416676369

Henry, C., Bigot, M., Masson, M., Siopi, E., and Dargel, A. A. (2018). Animal models of bipolar disorders: Towards new perspectives. *ANNALES MEDICO-PSYCHOLOGIQUES* 176, 200–204. doi: 10.1016/j.amp.2017.12.006

Herrington, J. D., Maddox, B. B., McVey, A. J., Franklin, M. E., Yerys, B. E., Miller, J. S., et al. (2017). Negative Valence in Autism Spectrum Disorder: The Relationship Between Amygdala Activity, Selective Attention, and Co-occurring Anxiety. *Biol Psychiatry Cogn Neurosci Neuroimaging* 2, 510–517. doi: 10.1016/j.bpsc.2017.03.009

Hofmann, S. G. (2014). Interpersonal Emotion Regulation Model of Mood and Anxiety Disorders. *Cognit Ther Res* 38, 483–492. doi: 10.1007/s10608-014-9620-1

Huey, E. D., Lee, S., Lieberman, J. A., Devanand, D. P., Brickman, A. M., Raymont, V., et al. (2016). Brain Regions Associated With Internalizing and Externalizing Psychiatric Symptoms in Patients With Penetrating Traumatic Brain Injury. *J Neuropsychiatry Clin Neurosci* 28, 104–111. doi: 10.1176/appi.neuropsych.15060150

Hur, J., Smith, J. F., DeYoung, K. A., Anderson, A. S., Kuang, J., Kim, H. C., et al. (2020). Anxiety and the Neurobiology of Temporally Uncertain Threat Anticipation. *J Neurosci* 40, 7949–7964. doi: 10.1523/JNEUROSCI.0704-20.2020

Ito, M., Miyamae, M., Yokoyama, C., Yamashita, Y., Ueno, O., Maruo, K., et al. (2019). Augmentation of Positive Valence System-Focused Cognitive Behavioral Therapy by Inaudible High-Frequency Sounds for Anhedonia: A Trial Protocol for a Pilot Study. *JAMA Netw Open* 2, e1915819. doi: 10.1001/jamanetworkopen.2019.15819

Jackson, M. G., and Robinson, E. S. J. (2022). The importance of a multidimensional approach to the preclinical study of major depressive disorder and apathy. *Emerg Top Life Sci* 6, 479–489. doi: 10.1042/ETLS20220004

Jagadapillai, R., Qiu, X., Ojha, K., Li, Z., El-Baz, A., Zou, S., et al. (2022). Potential Cross Talk between Autism Risk Genes and Neurovascular Molecules: A Pilot Study on Impact of Blood Brain Barrier Integrity. *Cells* 11. doi: 10.3390/cells11142211

Jagannath, V., Theodoridou, A., Gerstenberg, M., Franscini, M., Heekeren, K., Correll, C. U., et al. (2017). Prediction Analysis for Transition to Schizophrenia in Individuals at Clinical High Risk for Psychosis: The Relationship of DAO, DAOA, and NRG1 Variants with Negative Symptoms and Cognitive Deficits. *Front Psychiatry* 8, 292. doi: 10.3389/fpsyt.2017.00292

Javanbakht, A. (2018). A Theory of Everything: Overlapping Neurobiological Mechanisms of Psychotherapies of Fear and Anxiety Related Disorders. *Front Behav Neurosci* 12, 328. doi: 10.3389/fnbeh.2018.00328

Johnson, A., Rainville, J. R., Rivero-Ballon, G. N., Dhimitri, K., and Hodes, G. E. (2021). Testing the Limits of Sex Differences Using Variable Stress. *Neuroscience* 454, 72–84. doi: 10.1016/j.neuroscience.2019.12.034

Kalanthroff, E., Anholt, G. E., and Simpson, H. B. (2017). "Research domain criteria and OCD: An oxymoron?" in *Obsessive-compulsive disorder: Phenomenology, pathophysiology, and treatment*, ed. C. Pittenger (New York, NY: Oxford University Press), 689–701.

Kanchanatawan, B., Sriswasdi, S., Thika, S., Stoyanov, D., Sirivichayakul, S., Carvalho, A. F., et al. (2018). Towards a new classification of stable phase schizophrenia into major and simple neuro-cognitive psychosis: Results of unsupervised machine learning analysis. *J Eval Clin Pract* 24, 879–891. doi: 10.1111/jep.12945

Kangas, B. D., Der-Avakian, A., and Pizzagalli, D. A. (2022). Probabilistic Reinforcement Learning and Anhedonia. *Curr Top Behav Neurosci* 58, 355–377. doi: 10.1007/7854_2022_349

Karalunas, S. L., Antovich, D., Goh, P. K., Martel, M. M., Tipsord, J., Nousen, E. K., et al. (2021). Longitudinal network model of the co-development of temperament, executive functioning, and psychopathology symptoms in youth with and without ADHD. *Dev Psychopathol* 33, 1803–1820. doi: 10.1017/s0954579421000900

Kaye, J. T., Bradford, D. E., and Curtin, J. J. (2016). Psychometric properties of startle and corrugator response in NPU, affective picture viewing, and resting state tasks. *Psychophysiology* 53, 1241–1255. doi: 10.1111/psyp.12663

Khalsa, S. S., Adolphs, R., Cameron, O. G., Critchley, H. D., Davenport, P. W., Feinstein, J. S., et al. (2018). Interoception and Mental Health: A Roadmap. *Biol Psychiatry Cogn Neurosci Neuroimaging* 3, 501–513. doi: 10.1016/j.bpsc.2017.12.004

Kircanski, K., Zhang, S., Stringaris, A., Wiggins, J. L., Towbin, K. E., Pine, D. S., et al. (2017). Empirically derived patterns of psychiatric symptoms in youth: A latent profile analysis. *J Affect Disord* 216, 109–116. doi: 10.1016/j.jad.2016.09.016

Konicar, L., Prillinger, K., Klöbl, M., Lanzenberger, R., Antal, A., and Plener, P. L. (2022). Brain Stimulation for Emotion Regulation in Adolescents With Psychiatric Disorders: Study Protocol for a Clinical-Transdiagnostical, Randomized, Triple-Blinded and Sham-Controlled Neurotherapeutic Trial. *Front Psychiatry* 13, 840836. doi: 10.3389/fpsyt.2022.840836

Kramer, L., Sander, C., Bertsch, K., Gescher, D. M., Cackowski, S., Hegerl, U., et al. (2019). EEG-vigilance regulation in Borderline Personality Disorder. *Int J Psychophysiol* 139, 10–17. doi: 10.1016/j.ijpsycho.2019.02.007

Kujawa, A., and Burkhouse, K. L. (2017). Vulnerability to Depression in Youth: Advances from Affective Neuroscience. *Biol Psychiatry Cogn Neurosci Neuroimaging* 2, 28–37. doi: 10.1016/j.bpsc.2016.09.006

Kupferberg, A., Bicks, L., and Hasler, G. (2016). Social functioning in major depressive disorder. *Neurosci Biobehav Rev* 69, 313–332. doi: 10.1016/j.neubiorev.2016.07.002

Latzman, R. D., Young, L. J., and Hopkins, W. D. (2016). Displacement behaviors in chimpanzees (Pan troglodytes): A neurogenomics investigation of the RDoC Negative Valence Systems domain. *Psychophysiology* 53, 355–363. doi: 10.1111/psyp.12449

Lebowitz, E. R., Gee, D. G., Pine, D. S., and Silverman, W. K. (2018). Implications of the Research Domain Criteria project for childhood anxiety and its disorders. *Clin Psychol Rev* 64, 99–109. doi: 10.1016/j.cpr.2018.01.005

Lee, M., Aggen, S. H., Carney, D. M., Hahn, S., Moroney, E., Machlin, L., et al. (2017). Latent structure of negative valence measures in childhood. *Depress Anxiety* 34, 742–751. doi: 10.1002/da.22656

Leibold, N. K., van den Hove, D L A, Viechtbauer, W., Buchanan, G. F., Goossens, L., Lange, I., et al. (2016). CO2 exposure as translational cross-species experimental model for panic. *Transl Psychiatry* 6, e885. doi: 10.1038/tp.2016.162

Leventhal, A. M., Strong, D. R., Sussman, S., Kirkpatrick, M. G., Unger, J. B., Barrington-Trimis, J. L., et al. (2016). Psychiatric comorbidity in adolescent electronic and conventional cigarette use. *J Psychiatr Res* 73, 71–78. doi: 10.1016/j.jpsychires.2015.11.008

Loerinc, A. G. *Examining the effectiveness and feasibility of a self-guided version of positive affect treatment.* ProQuest Information & Learning.

Lowery-Gionta, E. G., DiBerto, J., Mazzone, C. M., and Kash, T. L. (2018). GABA neurons of the ventral periaqueductal gray area modulate behaviors associated with anxiety and conditioned fear. *Brain Struct Funct* 223, 3787–3799. doi: 10.1007/s00429-018-1724-z

Luoma, J. (2011). Member update on 2011 NIMH research priorities and advances. *Behav Ther (N Y N Y)* 34, 93–94.

Macpherson, T., and Hikida, T. (2019). Response to ‘mood and affect’. *Psychiatry Clin Neurosci* 73, 347. doi: 10.1111/pcn.12845

Masand, P. S., and Pae, C.-U. (2015). Cognitive dysfunction in psychiatric illness: A neglected domain. *J Clin Psychiatry* 76, e375-e377. doi: 10.4088/JCP.15com09780

Mason, B. L., Brown, E. S., and Croarkin, P. E. (2016). Historical Underpinnings of Bipolar Disorder Diagnostic Criteria. *Behav Sci (Basel)* 6. doi: 10.3390/bs6030014

McEvoy, P. M., Carleton, R. N., Correa, K., Shankman, S. A., and Shihata, S. (2019). “Intolerance of uncertainty,” in *The Cambridge handbook of anxiety and related disorders*, ed. B. O. Olatunji (New York, NY: Cambridge University Press), 196–226.

McIntyre, R. S., Johe, K., Rong, C., and Lee, Y. (2017). The neurogenic compound, NSI-189 phosphate: a novel multi-domain treatment capable of pro-cognitive and antidepressant effects. *Expert Opin Investig Drugs* 26, 767–770. doi: 10.1080/13543784.2017.1324847

Molnar, C. (2020). Michael J. Kozak (1952-2019). *Am Psychol* 75, 123. doi: 10.1037/amp0000518

Monteleone, A. M., Cascino, G., Ruzzi, V., Pellegrino, F., Carfagno, M., Raia, M., et al. (2020). Multiple levels assessment of the RDoC "system for social process" in Eating Disorders: Biological, emotional and cognitive responses to the Trier Social Stress Test. *J Psychiatr Res* 130, 160–166. doi: 10.1016/j.jpsychires.2020.07.039

Moreno-Montoya, M., Olmedo-Córdoba, M., and Martín-González, E. (2022). Negative valence system as a relevant domain in compulsivity: review in a preclinical model of compulsivity. *Emerg Top Life Sci* 6, 491–500. doi: 10.1042/ETLS20220005

Moser, J. S., Durbin, C. E., Patrick, C. J., and Schmidt, N. B. (2015). Combining neural and behavioral indicators in the assessment of internalizing psychopathology in children and adolescents. *J Clin Child Adolesc Psychol* 44, 329–340. doi: 10.1080/15374416.2013.865191

Muecke-Heim, I.-A. von, Ries, C., Urbina, L., and Deussing, J. M. (2021). P2X7R antagonists in chronic stress-based depression models: a review. *Eur Arch Psychiatry Clin Neurosci* 271, 1343–1358. doi: 10.1007/s00406-021-01306-3

Nguyen, T. N. B., Ely, B. A., Pick, D., Patel, M., Xie, H., Kim-Schulze, S., et al. (2022). Clenbuterol attenuates immune reaction to lipopolysaccharide and its relationship to anhedonia in adolescents. *Brain Behav Immun* 106, 89–99. doi: 10.1016/j.bbi.2022.07.163

Nicholson, J. R., and Sommer, B. (2018). The research domain criteria framework in drug discovery for neuropsychiatric diseases: focus on negative valence. *Brain Neurosci Adv* 2, 2398212818804030. doi: 10.1177/2398212818804030

Norrholm, S. D., Glover, E. M., Stevens, J. S., Fani, N., Galatzer-Levy, I. R., Bradley, B., et al. (2015). Fear load: The psychophysiological over-expression of fear as an intermediate phenotype associated with trauma reactions. *Int J Psychophysiol* 98, 270–275. doi: 10.1016/j.ijpsycho.2014.11.005

Northoff, G. (2020). “Brain and mind in psychiatry? Presuppositions of cognitive ontology,” in *Levels of analysis in psychopathology: Cross-disciplinary perspectives*, eds. K. S. Kendler, J. Parnas, and P. Zachar (New York, NY: Cambridge University Press), 78–85.

Oikonomidis, L., Santangelo, A. M., Shiba, Y., Clarke, F. H., Robbins, T. W., and Roberts, A. C. (2017). A dimensional approach to modeling symptoms of neuropsychiatric disorders in the marmoset monkey. *Dev Neurobiol* 77, 328–353. doi: 10.1002/dneu.22446

Owens, M., and Gibb, B. E. (2017). Brooding rumination and attentional biases in currently non-depressed individuals: an eye-tracking study. *Cogn Emot* 31, 1062–1069. doi: 10.1080/02699931.2016.1187116

Paris, J., and Bayes, A. (2019). “Differentiating bipolar II disorder from personality-based dysregulation disorders,” in *Bipolar II disorder: Modelling, measuring and managing., 3rd ed*, ed. G. Parker (New York, NY: Cambridge University Press), 77–90.

Park, S.-C., and Kim, Y.-K. (2019). A Novel Bio-Psychosocial-Behavioral Treatment Model of Panic Disorder. *Psychiatry Investig* 16, 4–15. doi: 10.30773/pi.2018.08.21.1

Paschali, M., Kiss, O., Zhao, Q., Adeli, E., Podhajsky, S., Müller-Oehring, E. M., et al. (2022). Detecting negative valence symptoms in adolescents based on longitudinal self-reports and behavioral assessments. *J Affect Disord* 312, 30–38. doi: 10.1016/j.jad.2022.06.002

Paschali, M., Zhao, Q., Adeli, E., and Pohl, K. M. (2022). Bridging the Gap between Deep Learning and Hypothesis-Driven Analysis via Permutation Testing. *Predict Intell Med* 13564, 13–23. doi: 10.1007/978-3-031-16919-9_2

Phan, K. L. (2015). “Neurobiology and neuroimaging of fear and anxiety circuitry,” in *Anxiety disorders: Translational perspectives on diagnosis and treatment*, eds. K. J. Ressler, D. S. Pine, and B. O. Rothbaum (New York, NY: Oxford University Press), 33–45.

Phillips, A. G., and Ahn, S. (2022). Anticipation: An Essential Feature of Anhedonia. *Curr Top Behav Neurosci* 58, 305–323. doi: 10.1007/7854_2022_317

Pollack, L. O. *Examination of non-facial emotion recognition in adolescents with anorexia nervosa.* ProQuest Information & Learning.

Premo, J. E., Liu, Y., Bilek, E. L., Phan, K. L., Monk, C. S., and Fitzgerald, K. D. (2020). Grant Report on Anxiety-CBT: Dimensional Brain Behavior Predictors of CBT Outcomes in Pediatric Anxiety. *J Psychiatr Brain Sci* 5. doi: 10.20900/jpbs.20200005

Puryear, C. B., Brooks, J., Tan, L., Smith, K., Li, Y., Cunningham, J., et al. (2020). Opioid receptor modulation of neural circuits in depression: What can be learned from preclinical data? *Neurosci Biobehav Rev* 108, 658–678. doi: 10.1016/j.neubiorev.2019.12.007

Putnam, K. T., Wilcox, M., Robertson-Blackmore, E., Sharkey, K., Bergink, V., Munk-Olsen, T., et al. (2017). 'Clinical phenotypes of perinatal depression and time of symptom onset: analysis of data from an international consortium': Correction. *Lancet Psychiatry* 4, 436. doi: 10.1016/S2215-0366(17)30213-4

Riboni, F. V., and Belzung, C. (2017). Stress and psychiatric disorders: from categorical to dimensional approaches. *CURRENT OPINION IN BEHAVIORAL SCIENCES* 14, 72–77. doi: 10.1016/j.cobeha.2016.12.011

Rodriguez, G., Moore, S. J., Neff, R. C., Glass, E. D., Stevenson, T. K., Stinnett, G. S., et al. (2020). Deficits across multiple behavioral domains align with susceptibility to stress in 129S1/SvImJ mice. *Neurobiol Stress* 13, 100262. doi: 10.1016/j.ynstr.2020.100262

Rohan, K. J., Franzen, P. L., Roeckelin, K. A., Siegle, G. J., Kolko, D. J., Postolache, T. T., et al. (2022). Elucidating treatment targets and mediators within a confirmatory efficacy trial: study protocol for a randomized controlled trial of cognitive-behavioral therapy vs. light therapy for winter depression. *Trials* 23, 383. doi: 10.1186/s13063-022-06330-9

Roma, P. G., Schneiderman, J. S., Schorn, J. M., Whiting, S. E., Landon, L. B., and Williams, T. J. (2021). Assessment of Spaceflight Medical Conditions' and Treatments' Potential Impacts on Behavioral Health and Performance. *Life Sci Space Res (Amst)* 30, 72–81. doi: 10.1016/j.lssr.2021.05.006

Rondon, M. B., and Stewart, D. E. (2017). Disentangling the heterogeneity of perinatal depression. *Lancet Psychiatry* 4, 432–433. doi: 10.1016/S2215-0366(17)30192-X

Ross, C. A., and Margolis, R. L. (2019). Research Domain Criteria: Strengths, Weaknesses, and Potential Alternatives for Future Psychiatric Research. *Mol Neuropsychiatry* 5, 218–236. doi: 10.1159/000501797

Rosso, I. M., Dillon, D. G., Pizzagalli, D. A., and Rauch, S. L. (2015). "Translational perspectives on anxiety disorders and the research domain criteria construct of potential threat," in *Anxiety disorders: Translational perspectives on diagnosis and treatment*, eds. K. J. Ressler, D. S. Pine, and B. O. Rothbaum (New York, NY: Oxford University Press), 17–29.

Santana-Santana, M., Bayascas, J.-R., and Giménez-Llort, L. (2021). Fine-Tuning the PI3K/Akt Signaling Pathway Intensity by Sex and Genotype-Load: Sex-Dependent Homozygotic Threshold for Somatic Growth but Feminization of Anxious Phenotype in Middle-Aged PDK1 K465E Knock-In and Heterozygous Mice. *Biomedicines* 9. doi: 10.3390/biomedicines9070747

Schettino, M., Ghezzi, V., Ang, Y.-S., Duda, J. M., Fagioli, S., Mennin, D. S., et al. (2021). Perseverative Cognition in the Positive Valence Systems: An Experimental and Ecological Investigation. *Brain Sci* 11. doi: 10.3390/brainsci11050585

Schmidt, U., and Vermetten, E. (2018). Integrating NIMH Research Domain Criteria (RDoC) into PTSD Research. *Curr Top Behav Neurosci* 38, 69–91. doi: 10.1007/7854_2017_1

Seager, I., Rowley, A. M., and Ehrenreich-May, J. (2014). Targeting Common Factors Across Anxiety and Depression Using the Unified Protocol for the Treatment of Emotional Disorders in Adolescents. *JOURNAL OF RATIONAL-EMOTIVE AND COGNITIVE-BEHAVIOR THERAPY* 32, 67–83. doi: 10.1007/s10942-014-0185-4

Seok, D., Smyk, N., Jaskir, M., Cook, P., Elliott, M., Girelli, T., et al. (2020). Dimensional connectomics of anxious misery, a human connectome study related to human disease: Overview of protocol and data quality. *Neuroimage Clin* 28, 102489. doi: 10.1016/j.nicl.2020.102489

Severus, E., Ebner-Priemer, U., Beier, F., Mühlbauer, E., Ritter, P., Hill, H., et al. (2019). Ambulatory monitoring and digital phenotyping in the diagnostics and treatment of bipolar disorders. *Nervenarzt* 90, 1215–1220. doi: 10.1007/s00115-019-00816-9

Shalev, I. (2018). Use of a Self-Regulation Failure Framework and the NIMH Research Domain Criterion (RDoC) to Understand the Problem of Procrastination. *Front Psychiatry* 9. doi: 10.3389/fpsyt.2018.00213

Shankman, S. A., Katz, A. C., and Langenecker, S. A. (2016). Taking an RDoC lens to the study of panic disorder: A commentary on Hamm et al. and other thoughts on RDoC. *Psychophysiology* 53, 328–331. doi: 10.1111/psyp.12590

Sill, J., Popov, T., Schauer, M., and Elbert, T. (2020). Rapid brain responses to affective pictures indicate dimensions of trauma-related psychopathology in adolescents. *Psychophysiology* 57, e13353. doi: 10.1111/psyp.13353

Smith, N. J., Markowitz, S. Y., Hoffman, A. N., and Fanselow, M. S. (2022). Adaptation of Threat Responses Within the Negative Valence Framework. *Front Syst Neurosci* 16, 886771. doi: 10.3389/fnsys.2022.886771

Stewart, J. G., Polanco-Roman, L., Duarte, C. S., and Auerbach, R. P. (2019). Neurocognitive Processes Implicated in Adolescent Suicidal Thoughts and Behaviors: Applying an RDoC Framework for Conceptualizing Risk. *Curr Behav Neurosci Rep* 6, 188–196. doi: 10.1007/s40473-019-00194-1

Stringaris, A., Vidal-Ribas Belil, P., Artiges, E., Lemaitre, H., Gollier-Briant, F., Wolke, S., et al. (2015). The brain's response to reward anticipation and depression in adolescence: Dimensionality, specificity, and longitudinal predictions in a community-based sample. *Am J Psychiatry* 173, 1215–1223. doi: 10.1037/t01528-000;

Tozzi, L., Staveland, B., Holt-Gosselin, B., Chesnut, M., Chang, S. E., Choi, D., et al. (2020). The human connectome project for disordered emotional states: Protocol and rationale for a research domain criteria study of brain connectivity in young adult anxiety and depression. *Neuroimage* 214, 116715. doi: 10.1016/j.neuroimage.2020.116715

Trucco, E. M., Villafuerte, S., Hussong, A., Burmeister, M., and Zucker, R. A. (2018). Biological underpinnings of an internalizing pathway to alcohol, cigarette, and marijuana use. *J Abnorm Psychol* 127, 79–91. doi: 10.1037/abn0000310

Vaidyanathan, U., and Pacheco, J. (2017). Research domain criteria constructs: Integrative reviews and empirical perspectives. *J Affect Disord* 216, 1–2. doi: 10.1016/j.jad.2017.05.028

Vargas, T. G., and Mittal, V. A. (2021). Testing whether implicit emotion regulation mediates the association between discrimination and symptoms of psychopathology in late childhood: An RDoC perspective. *Dev Psychopathol* 33, 1634–1647. doi: 10.1017/S0954579421000638

Victor, T. A., Khalsa, S. S., Simmons, W. K., Feinstein, J. S., Savitz, J., Aupperle, R. L., et al. (2018). Tulsa 1000: a naturalistic study protocol for multilevel assessment and outcome prediction in a large psychiatric sample. *BMJ Open* 8, e016620. doi: 10.1136/bmjopen-2017-016620

Vogel, A. C., Tillman, R., El-Sayed, N. M., Jackson, J. J., Perlman, S. B., Barch, D. M., et al. (2021). Trajectory of emotion dysregulation in positive and negative affect across childhood predicts adolescent emotion dysregulation and overall functioning. *Dev Psychopathol* 33, 1722–1733. doi: 10.1017/s0954579421000705

Walther, S., Bernard, J. A., Mittal, V. A., and Shankman, S. A. (2019). The utility of an RDoC motor domain to understand psychomotor symptoms in depression. *Psychol Med* 49, 212–216. doi: 10.1017/S0033291718003033

Warren, S. L., Zhang, Y., Duberg, K., Mistry, P., Cai, W., Qin, S., et al. (2020). Anxiety and Stress Alter Decision-Making Dynamics and Causal Amygdala-Dorsolateral Prefrontal Cortex Circuits During Emotion Regulation in Children. *Biol Psychiatry* 88, 576–586. doi: 10.1016/j.biopsych.2020.02.011

Weinberg, A., Meyer, A., Hale-Rude, E., Perlman, G., Kotov, R., Klein, D. N., et al. (2016). Error-related negativity (ERN) and sustained threat: Conceptual framework and empirical evaluation in an adolescent sample. *Psychophysiology* 53, 372–385. doi: 10.1111/psyp.12538

Wielgosz, J., Goldberg, S. B., Kral, T. R. A., Dunne, J. D., and Davidson, R. J. (2019). Mindfulness Meditation and Psychopathology. *Annu Rev Clin Psychol* 15, 285–316. doi: 10.1146/annurev-clinpsy-021815-093423

Williams, L. M., Goldstein-Piekarski, A. N., Chowdhry, N., Grisanzio, K. A., Haug, N. A., Samara, Z., et al. (2016). Developing a clinical translational neuroscience taxonomy for anxiety and mood disorder: protocol for the baseline-follow up Research domain criteria Anxiety and Depression ("RAD") project. *BMC Psychiatry* 16, 68. doi: 10.1186/s12888-016-0771-3

Yang, Z., Oathes, D. J., Linn, K. A., Bruce, S. E., Satterthwaite, T. D., Cook, P. A., et al. (2018). Cognitive Behavioral Therapy Is Associated With Enhanced Cognitive Control Network Activity in Major Depression and Posttraumatic Stress Disorder. *Biol Psychiatry Cogn Neurosci Neuroimaging* 3, 311–319. doi: 10.1016/j.bpsc.2017.12.006

Young, J. W., Winstanley, C. A., Brady, A. M., and Hall, F. S. (2017). Research Domain Criteria versus DSM V: How does this debate affect attempts to model corticostriatal dysfunction in animals? *Neurosci Biobehav Rev* 76, 301–316. doi: 10.1016/j.neubiorev.2016.10.029

Zainal, N. H., Newman, M. G., and Hong, R. Y. (2021). Cross-Cultural and Gender Invariance of Transdiagnostic Processes in the United States and Singapore. *Assessment* 28, 485–502. doi: 10.1177/1073191119869832

Zhou, A. J., Lee, Y., Salvadore, G., Hsu, B., Fonseka, T. M., Kennedy, S. H., et al. (2017). Sirukumab: A Potential Treatment for Mood Disorders? *Adv Ther* 34, 78–90. doi: 10.1007/s12325-016-0455-x

Zilverstand, A., Parvaz, M. A., and Goldstein, R. Z. (2017). Neuroimaging cognitive reappraisal in clinical populations to define neural targets for enhancing emotion regulation. A systematic review. *Neuroimage* 151, 105–116. doi: 10.1016/j.neuroimage.2016.06.009

2 Sources excluded following full-text review

Alexopoulos, G. S., and Arean, P. (2014). A model for streamlining psychotherapy in the RDoC era: the example of 'Engage'. *Mol Psychiatry* 19, 14–19. doi: 10.1038/mp.2013.150

Arditte, K. A. *Cognitive vulnerability for social anxiety and depression: A transdiagnostic investigation of repetitive negative thinkers.* ProQuest Information & Learning.

Bauer, E. A., Wilson, K. A., Phan, K. L., Shankman, S. A., and MacNamara, A. (2023). A Neurobiological Profile Underlying Comorbidity Load and Prospective Increases in Dysphoria in a Focal Fear Sample. *Biol Psychiatry* 93, 352–361. doi: 10.1016/j.biopsych.2022.08.009

Bedwell, J. S., Gooding, D. C., Chan, C. C., and Trachik, B. J. (2014). Anhedonia in the age of RDoC. *Schizophr Res* 160, 226–227. doi: 10.1016/j.schres.2014.10.028

Broome, M. R., He, Z., Iftikhar, M., Eyden, J., and Marwaha, S. (2015). Neurobiological and behavioural studies of affective instability in clinical populations: a systematic review. *Neurosci Biobehav Rev* 51, 243–254. doi: 10.1016/j.neubiorev.2015.01.021

Gros, D. F. (2015). Design challenges in transdiagnostic psychotherapy research: Comparing Transdiagnostic Behavior Therapy (TBT) to existing evidence-based psychotherapy in veterans with affective disorders. *Contemp Clin Trials* 43, 114–119. doi: 10.1016/j.cct.2015.05.011

Gururajan, A., Clarke, G., Dinan, T. G., and Cryan, J. F. (2016). Molecular biomarkers of depression. *Neurosci Biobehav Rev* 64, 101–133. doi: 10.1016/j.neubiorev.2016.02.011

Hasratian, A. M., Meuret, A. E., Chmielewski, M., and Ritz, T. (2022). An Examination of the RDoC Negative Valence Systems Domain Constructs and the Self-Reports Unit of Analysis. *Behav Ther* 53, 1092–1108. doi: 10.1016/j.beth.2022.04.009

Jauhar, S., Nour, M. M., Veronese, M., Rogdaki, M., Bonoldi, I., Azis, M., et al. (2017). A Test of the Transdiagnostic Dopamine Hypothesis of Psychosis Using Positron Emission Tomographic Imaging in Bipolar Affective Disorder and Schizophrenia. *JAMA Psychiatry* 74, 1206–1213. doi: 10.1001/jamapsychiatry.2017.2943

Kelly, J. R., Gillan, C. M., Prenderville, J., Kelly, C., Harkin, A., Clarke, G., et al. (2021). Psychedelic Therapy's Transdiagnostic Effects: A Research Domain Criteria (RDoC) Perspective. *Front Psychiatry* 12, 800072. doi: 10.3389/fpsyt.2021.800072

Kim, J. U., Bessette, K. L., Westlund-Schreiner, M., Pocius, S., Dillahunt, A. K., Frandsen, S., et al. (2022). Relations of gray matter volume to dimensional measures of cognition and affect in mood disorders. *Cortex* 156, 57–70. doi: 10.1016/j.cortex.2022.06.019

Kirsch, J. L., and Ehlers, S. L. (2022). Factor Analysis of the Beck Depression Inventory-II and Long-Term Hematopoietic Stem Cell Transplantation Survival Using the Research Domain Criteria Framework. *Transplant Cell Ther*. doi: 10.1016/j.jtct.2022.12.012

Kiyar, M., Lommen, M. J. J., Krebs, R. M., Daniels, J. K., and Mueller, S. C. (2021). Reward prospect improves inhibitory control in female university students with a history of childhood sexual and physical abuse. *J Behav Ther Exp Psychiatry* 71, 101629. doi: 10.1016/j.jbtep.2020.101629

Klemanski, D. H., Curtiss, J., McLaughlin, K. A., and Nolen-Hoeksema, S. (2017). Emotion Regulation and the Transdiagnostic Role of Repetitive Negative Thinking in Adolescents with Social Anxiety and Depression. *Cognit Ther Res* 41, 206–219. doi: 10.1007/s10608-016-9817-6

Kong, J., Xu, H., Ji, X., Zhang, J., Yang, H., Zhang, Y., et al. (2020). Attribution Retraining Group Therapy and SSRIs Affect Differing Facets of Anxiety Among Chinese Patients with Various Diagnoses: A Single-Center, Prospective Study. *PSYCHIATRY AND CLINICAL PSYCHOPHARMACOLOGY* 30, 97–106. doi: 10.5455/PCP.20200530013456

Lang, P. J., McTeague, L. M., and Bradley, M. M. (2017). The Psychophysiology of Anxiety and Mood Disorders. *ZEITSCHRIFT FUR PSYCHOLOGIE-JOURNAL OF PSYCHOLOGY* 225, 175–188. doi: 10.1027/2151-2604/a000302

Langenecker, S. A., Mickey, B. J., Eichhammer, P., Sen, S., Elverman, K. H., Kennedy, S. E., et al. (2019). Cognitive Control as a 5-HT(1A)-Based Domain That Is Disrupted in Major Depressive Disorder. *Front Psychol* 10, 691. doi: 10.3389/fpsyg.2019.00691

Li, J. J., Zhang, Q., Wang, Z., and Lu, Q. (2022). Research Domain Criteria (RDoC) mechanisms of transdiagnostic polygenic risk for trajectories of depression: From early adolescence to adulthood. *J Psychopathol Clin Sci* 131, 567–574. doi: 10.1037/abn0000659

Liu, H., Lieberman, L., Stevens, E. S., Auerbach, R. P., and Shankman, S. A. (2017). Using a cultural and RDoC framework to conceptualize anxiety in Asian Americans. *J Anxiety Disord* 48, 63–69. doi: 10.1016/j.janxdis.2016.09.006

Liu, Y., Meng, J., Wang, K., Zhuang, K., Chen, Q., Yang, W., et al. (2021). Morphometry of the Hippocampus Across the Adult Life-Span in Patients with Depressive Disorders: Association with Neuroticism. *Brain Topogr* 34, 587–597. doi: 10.1007/s10548-021-00846-0

Lu, S., Zhang, Y., Liu, T., Leung, D. K. Y., Kwok, W.-W., Luo, H., et al. (2022). Associations between depressive symptom clusters and care utilization and costs among community-dwelling older adults. *Int J Geriatr Psychiatry* 37. doi: 10.1002/gps.5636

Magaraggia, I., Kuiperes, Z., and Schreiber, R. (2021). Improving cognitive functioning in major depressive disorder with psychedelics: A dimensional approach. *Neurobiol Learn Mem* 183, 107467. doi: 10.1016/j.nlm.2021.107467

Mareckova, K., Holsen, L., Admon, R., Whitfield-Gabrieli, S., Seidman, L. J., Buka, S. L., et al. (2017). Neural - hormonal responses to negative affective stimuli: Impact of dysphoric mood and sex. *J Affect Disord* 222, 88–97. doi: 10.1016/j.jad.2017.06.050

Mareckova, K., Holsen, L. M., Admon, R., Makris, N., Seidman, L., Buka, S., et al. (2016). Brain activity and connectivity in response to negative affective stimuli: Impact of dysphoric mood and sex across diagnoses. *Hum Brain Mapp* 37, 3733–3744. doi: 10.1002/hbm.23271

McKay, D., and Tolin, D. F. (2017). Empirically supported psychological treatments and the Research Domain Criteria (RDoC). *J Affect Disord* 216, 78–88. doi: 10.1016/j.jad.2016.10.018

Park, S.-C., and Kim, Y.-K. (2021). Challenges and Strategies for Current Classifications of Depressive Disorders: Proposal for Future Diagnostic Standards. *Adv Exp Med Biol* 1305, 103–116. doi: 10.1007/978-981-33-6044-0_7

Podlogar, M. C., Rogers, M. L., Stanley, I. H., Hom, M. A., Chiurliza, B., and Joiner, T. E. (2018). Anxiety, depression, and the suicidal spectrum: a latent class analysis of overlapping and distinctive features. *Cogn Emot* 32, 1464–1477. doi: 10.1080/02699931.2017.1303452

Portugal, L. C. L., Schrouff, J., Stiffler, R., Bertocci, M., Bebko, G., Chase, H., et al. (2019). Predicting anxiety from wholebrain activity patterns to emotional faces in young adults: a machine learning approach. *Neuroimage Clin* 23, 101813. doi: 10.1016/j.nicl.2019.101813

Putnam, K. T., Wilcox, M., Robertson-Blackmore, E., Sharkey, K., Bergink, V., Munk-Olsen, T., et al. (2017). Clinical phenotypes of perinatal depression and time of symptom onset: analysis of data from an international consortium. *Lancet Psychiatry* 4, 477–485. doi: 10.1016/S2215-0366(17)30136-0

Rzepa, E., and McCabe, C. (2019). Dimensional anhedonia and the adolescent brain: reward and aversion anticipation, effort and consummation. *BJPsych Open* 5, e99. doi: 10.1192/bjo.2019.68

Seok, D., Beer, J., Jaskir, M., Smyk, N., Jaganjac, A., Makhoul, W., et al. (2022). Differential Impact of Anxious Misery Psychopathology on Multiple Representations of the Functional Connectome. *Biol Psychiatry Glob Open Sci* 2, 489–499. doi: 10.1016/j.bpsgos.2021.11.004

Shackman, A. J., and Fox, A. S. (2016). Contributions of the Central Extended Amygdala to Fear and Anxiety. *J Neurosci* 36, 8050–8063. doi: 10.1523/JNEUROSCI.0982-16.2016

Sharp, C., Kim, S., Herman, L., Pane, H., Reuter, T., and Strathearn, L. (2014). Major depression in mothers predicts reduced ventral striatum activation in adolescent female offspring with and without depression. *J Abnorm Psychol* 123, 298–309. doi: 10.1037/a0036191

Sharp, P. B., Miller, G. A., and Heller, W. (2015). Transdiagnostic dimensions of anxiety: Neural mechanisms, executive functions, and new directions. *Int J Psychophysiol* 98, 365–377. doi: 10.1016/j.ijpsycho.2015.07.001

Souza, A. M. F. L. P. de, Nonohay, R. G. de, and Gauer, G. (2018). Frustrating feedback effects on Error-Related Negativity (ERN) as a dimensional marker of negative affect. *CLINICAL NEUROPSYCHIATRY* 15, 275–283. doi: 10.1037/t06070-000.

Sumner, J. A., Powers, A., Jovanovic, T., and Koenen, K. C. (2016). Genetic influences on the neural and physiological bases of acute threat: A research domain criteria (RDoC) perspective. *Am J Med Genet B Neuropsychiatr Genet* 171B, 44–64. doi: 10.1002/ajmg.b.32384

Swardfager, W., Rosenblat, J. D., Benlamri, M., and McIntyre, R. S. (2016). Mapping inflammation onto mood: Inflammatory mediators of anhedonia. *Neurosci Biobehav Rev* 64, 148–166. doi: 10.1016/j.neubiorev.2016.02.017

Talati, A., van Dijk, M. T., Pan, L., Hao, X., Wang, Z., Gameroff, M., et al. (2022). Putamen Structure and Function in Familial Risk for Depression: A Multimodal Imaging Study. *Biol Psychiatry* 92, 932–941. doi: 10.1016/j.biopsych.2022.06.035

van Meter, A. R., and Youngstrom, E. A. (2015). A tale of two diatheses: Temperament, BIS, and BAS as risk factors for mood disorder. *J Affect Disord* 180, 170–178. doi: 10.1016/j.jad.2015.03.053

Walter, H., Daniels, A., and Wellan, S. A. (2021). Positive cognitive neuroscience : Positive valence systems of the Research Domain Criteria initiative. *Nervenarzt* 92, 878–891. doi: 10.1007/s00115-021-01167-0

Ward, J., Strawbridge, R. J., Bailey, M. E. S., Graham, N., Ferguson, A., Lyall, D. M., et al. (2017). Genome-wide analysis in UK Biobank identifies four loci associated with mood instability and genetic correlation with major depressive disorder, anxiety disorder and schizophrenia. *Transl Psychiatry* 7, 1264. doi: 10.1038/s41398-017-0012-7

Watson, D., Stanton, K., and Clark, L. A. (2017). Self-report indicators of negative valence constructs within the research domain criteria (RDoC): A critical review. *J Affect Disord* 216, 58–69. doi: 10.1016/j.jad.2016.09.065

Wei, M., and Roodenrys, S. (2021). A scoping review on the extent and nature of anxiety-related research within the research domain criteria (RDoC) framework: Limited coverage using non-disorder-specific search terms. *New Ideas Psychol* 63. doi: 10.1016/j.newideapsych.2021.100901

Wu, M.-J., Mwangi, B., Bauer, I. E., Passos, I. C., Sanches, M., Zunta-Soares, G. B., et al. (2017). Identification and individualized prediction of clinical phenotypes in bipolar disorders using neurocognitive data, neuroimaging scans and machine learning. *Neuroimage* 145, 254–264. doi: 10.1016/j.neuroimage.2016.02.016

Xie, J., Fang, P., Zhang, Z., Luo, R., and Dai, B. (2021). Behavioral Inhibition/Activation Systems and Depression Among Females With Substance Use Disorder: The Mediating Role of Intolerance of Uncertainty and Anhedonia. *Front Psychiatry* 12, 644882. doi: 10.3389/fpsyt.2021.644882

Yaseen, Z. S., Galynker, I. I., Briggs, J., Freed, R. D., and Gabbay, V. (2016). Functional domains as correlates of suicidality among psychiatric inpatients. *J Affect Disord* 203, 77–83. doi: 10.1016/j.jad.2016.05.066

3 Sources included in synthesis

Alexopoulos, G. S., Raue, P. J., Gunning, F., Kiosses, D. N., Kanellopoulos, D., Pollari, C., et al. (2016). "Engage" Therapy: Behavioral Activation and Improvement of Late-Life Major Depression. *Am J Geriatr Psychiatry* 24, 320–326. doi: 10.1016/j.jagp.2015.11.006

Alexopoulos, G. S., Raue, P. J., Kiosses, D. N., Seirup, J. K., Banerjee, S., and Arean, P. A. (2015). Comparing engage with PST in late-life major depression: a preliminary report. *Am J Geriatr Psychiatry* 23, 506–513. doi: 10.1016/j.jagp.2014.06.008

Barch, D. M., Pagliaccio, D., and Luking, K. (2016). Mechanisms Underlying Motivational Deficits in Psychopathology: Similarities and Differences in Depression and Schizophrenia. *Curr Top Behav Neurosci* 27, 411–449. doi: 10.1007/7854_2015_376

Baskin-Sommers, A. R., and Foti, D. (2015). Abnormal reward functioning across substance use disorders and major depressive disorder: Considering reward as a transdiagnostic mechanism. *Int J Psychophysiol* 98, 227–239. doi: 10.1016/j.ijpsycho.2015.01.011

Boecker, L., and Pauli, P. (2019). Affective startle modulation and psychopathology: Implications for appetitive and defensive brain systems. *Neurosci Biobehav Rev* 103, 230–266. doi: 10.1016/j.neubiorev.2019.05.019

Cochran, A. L., Pingeton, B. C., Goodman, S. H., Laurent, H., Rathouz, P. J., Newport, D. J., et al. (2020). A transdiagnostic approach to conceptualizing depression across the perinatal period in a high-risk sample. *J Abnorm Psychol* 129, 689–700. doi: 10.1037/abn0000612

Ellingson, J. M., Richmond-Rakerd, L. S., Statham, D. J., Martin, N. G., and Slutske, W. S. (2016). Most of the genetic covariation between major depressive and alcohol use disorders is explained by trait measures of negative emotionality and behavioral control. *Psychol Med* 46, 2919–2930. doi: 10.1017/S0033291716001525

Ethridge, P., Freeman, C., Sandre, A., Banica, I., Dirks, M. A., and Weinberg, A. (2021). Intergenerational transmission of depression risk: Mothers' neural response to reward and history of depression are associated with daughters' neural response to reward across adolescence. *J Abnorm Psychol*. doi: 10.1037/abn0000662

Fettes, P., Schulze, L., and Downar, J. (2017). Cortico-Striatal-Thalamic Loop Circuits of the Orbitofrontal Cortex: Promising Therapeutic Targets in Psychiatric Illness. *Front Syst Neurosci* 11, 25. doi: 10.3389/fnsys.2017.00025

Förstner, B. R., Tschorn, M., Reinoso-Schiller, N., Maričić, L. M., Röcher, E., Kalman, J. L., et al. (2022). Mapping Research Domain Criteria using a transdiagnostic mini-RDoC assessment in mental disorders: a confirmatory factor analysis. *Eur Arch Psychiatry Clin Neurosci*. doi: 10.1007/s00406-022-01440-6

Gibb, B. E., McGeary, J. E., and Beevers, C. G. (2016). Attentional biases to emotional stimuli: Key components of the RDoC constructs of sustained threat and loss. *Am J Med Genet B Neuropsychiatr Genet* 171B, 65–80. doi: 10.1002/ajmg.b.32383

Gruber, J., Mennin, D. S., Fields, A., Purcell, A., and Murray, G. (2015). Heart rate variability as a potential indicator of positive valence system disturbance: A proof of concept investigation. *Int J Psychophysiol* 98, 240–248. doi: 10.1016/j.ijpsycho.2015.08.005

Guineau, M. G., Ikani, N., Rinck, M., Collard, R. M., van Eijndhoven, P., Tendolkar, I., et al. (2022). Anhedonia as a transdiagnostic symptom across psychological disorders: a network approach. *Psychol Med*, 1–12. doi: 10.1017/S0033291722000575

Gunzler, D., Sehgal, A. R., Kauffman, K., Davey, C. H., Dolata, J., Figueroa, M., et al. (2020). Identify depressive phenotypes by applying RDOC domains to the PHQ-9. *Psychiatry Res* 286, 112872. doi: 10.1016/j.psychres.2020.112872

Hamm, A. O., Richter, J., Pané-Farré, C., Westphal, D., Wittchen, H.-U., Vossbeck-Elsebusch, A. N., et al. (2016). Panic disorder with agoraphobia from a behavioral neuroscience perspective: Applying the research principles formulated by the Research Domain Criteria (RDoC) initiative. *Psychophysiology* 53, 312–322. doi: 10.1111/psyp.12553

Janiri, D., Moser, D. A., Doucet, G. E., Luber, M. J., Rasgon, A., Lee, W. H., et al. (2020). Shared Neural Phenotypes for Mood and Anxiety Disorders: A Meta-analysis of 226 Task-Related Functional Imaging Studies. *JAMA Psychiatry* 77, 172–179. doi: 10.1001/jamapsychiatry.2019.3351

Khazanov, G. K., Ruscio, A. M., and Forbes, C. N. (2020). The Positive Valence Systems Scale: Development and Validation. *Assessment* 27, 1045–1069. doi: 10.1177/1073191119869836

Klumpp, H., and Shankman, S. A. (2018). Using Event-Related Potentials and Startle to Evaluate Time Course in Anxiety and Depression. *Biol Psychiatry Cogn Neurosci Neuroimaging* 3, 10–18. doi: 10.1016/j.bpsc.2017.09.004

Lang, P. J., Herring, D. R., Duncan, C., Richter, J., Sege, C. T., Weymar, M., et al. (2018). The Startle-Evoked Potential: Negative Affect and Severity of Pathology in Anxiety/Mood Disorders. *Biol Psychiatry Cogn Neurosci Neuroimaging* 3, 626–634. doi: 10.1016/j.bpsc.2017.07.006

Lang, P. J., McTeague, L. M., and Bradley, M. M. (2016). RDoC, DSM, and the reflex physiology of fear: A biodimensional analysis of the anxiety disorders spectrum. *Psychophysiology* 53, 336–347. doi: 10.1111/psyp.12462

Langenecker, S. A., Jacobs, R. H., and Passarotti, A. M. (2014). Current Neural and Behavioral Dimensional Constructs across Mood Disorders. *Curr Behav Neurosci Rep* 1, 144–153. doi: 10.1007/s40473-014-0018-x

Langenecker, S. A., Westlund Schreiner, M., Thomas, L. R., Bessette, K. L., DelDonno, S. R., Jenkins, L. M., et al. (2022). Using Network Parcels and Resting-State Networks to Estimate Correlates of Mood Disorder and Related Research Domain Criteria Constructs of Reward Responsiveness and Inhibitory Control. *Biol Psychiatry Cogn Neurosci Neuroimaging* 7, 76–84. doi: 10.1016/j.bpsc.2021.06.014

MacNamara, A., Klumpp, H., Kennedy, A. E., Langenecker, S. A., and Phan, K. L. (2017). Transdiagnostic neural correlates of affective face processing in anxiety and depression. *Depress Anxiety* 34, 621–631. doi: 10.1002/da.22631

McTeague, L. M., Rosenberg, B. M., Lopez, J. W., Carreon, D. M., Huemer, J., Jiang, Y., et al. (2020). Identification of Common Neural Circuit Disruptions in Emotional Processing Across Psychiatric Disorders. *Am J Psychiatry* 177, 411–421. doi: 10.1176/appi.ajp.2019.18111271

Medeiros, G. C., Rush, A. J., Jha, M., Carmody, T., Furman, J. L., Czysz, A. H., et al. (2020). Positive and negative valence systems in major depression have distinct clinical features, response to antidepressants, and relationships with immunomarkers. *Depress Anxiety* 37, 771–783. doi: 10.1002/da.23006

Nakonezny, P. A., Morris, D. W., Greer, T. L., Byerly, M. J., Carmody, T. J., Grannemann, B. D., et al. (2015). Evaluation of anhedonia with the Snaith-Hamilton Pleasure Scale (SHAPS) in adult outpatients with major depressive disorder. *J Psychiatr Res* 65, 124–130. doi: 10.1016/j.jpsychires.2015.03.010

Nusslock, R., and Alloy, L. B. (2017). Reward processing and mood-related symptoms: An RDoC and translational neuroscience perspective. *J Affect Disord* 216, 3–16. doi: 10.1016/j.jad.2017.02.001

Nusslock, R., Walden, K., and Harmon-Jones, E. (2015). Asymmetrical frontal cortical activity associated with differential risk for mood and anxiety disorder symptoms: An RDoC perspective. *Int J Psychophysiol* 98, 249–261. doi: 10.1016/j.ijpsycho.2015.06.004

Olino, T. M., McMakin, D. L., and Forbes, E. E. (2018). Toward an empirical multidimensional structure of anhedonia, reward sensitivity, and positive emotionality: An exploratory factor analytic study. *Assessment* 25, 679–690. doi: 10.1177/1073191116680291

Paulus, M. P., Stein, M. B., Craske, M. G., Bookheimer, S., Taylor, C. T., Simmons, A. N., et al. (2017). Latent variable analysis of positive and negative valence processing focused on symptom and behavioral units of analysis in mood and anxiety disorders. *J Affect Disord* 216, 17–29. doi: 10.1016/j.jad.2016.12.046

Peng, Y., Knotts, J. D., Taylor, C. T., Craske, M. G., Stein, M. B., Bookheimer, S., et al. (2021). Failure to Identify Robust Latent Variables of Positive or Negative Valence Processing Across Units of Analysis. *Biol Psychiatry Cogn Neurosci Neuroimaging* 6, 518–526. doi: 10.1016/j.bpsc.2020.12.005

Ross, R. A., Foster, S. L., and Ionescu, D. F. (2017). The Role of Chronic Stress in Anxious Depression. *Chronic Stress (Thousand Oaks)* 1, 2470547016689472. doi: 10.1177/2470547016689472

Sambuco, N., Bradley, M., Herring, D., Hillbrandt, K., and Lang, P. J. (2020). Transdiagnostic trauma severity in anxiety and mood disorders: Functional brain activity during emotional scene processing. *Psychophysiology* 57, e13349. doi: 10.1111/psyp.13349

Savage, J. E., Sawyers, C., Roberson-Nay, R., and Hettema, J. M. (2017). The genetics of anxiety-related negative valence system traits. *Am J Med Genet B Neuropsychiatr Genet* 174, 156–177. doi: 10.1002/ajmg.b.32459

Silveira, Érico de M Jr, and Kauer-Sant'Anna, M. (2015). Rumination in bipolar disorder: a systematic review. *Braz J Psychiatry* 37, 256–263. doi: 10.1590/1516-4446-2014-1556

Swope, A. K., Fredrick, J. W., Becker, S. P., Burns, G. L., Garner, A. A., Jarrett, M. A., et al. (2020). Sluggish cognitive tempo and positive valence systems: Unique relations with greater reward valuation but less willingness to work. *J Affect Disord* 261, 131–138. doi: 10.1016/j.jad.2019.10.006

Taylor, W. D., Zald, D. H., Felger, J. C., Christman, S., Claassen, D. O., Horga, G., et al. (2022). Influences of dopaminergic system dysfunction on late-life depression. *Mol Psychiatry* 27, 180–191. doi: 10.1038/s41380-021-01265-0

Terbeck, S., Akkus, F., Chesterman, L. P., and Hasler, G. (2015). The role of metabotropic glutamate receptor 5 in the pathogenesis of mood disorders and addiction: combining preclinical evidence with human Positron Emission Tomography (PET) studies. *Front Neurosci* 9, 86. doi: 10.3389/fnins.2015.00086

Toups, M., Carmody, T., Greer, T., Rethorst, C., Grannemann, B., and Trivedi, M. H. (2017). Exercise is an effective treatment for positive valence symptoms in major depression. *J Affect Disord* 209, 188–194. doi: 10.1016/j.jad.2016.08.058

Trøstheim, M., Eikemo, M., Meir, R., Hansen, I., Paul, E., Kroll, S. L., et al. (2020). Assessment of Anhedonia in Adults With and Without Mental Illness: A Systematic Review and Meta-analysis. *JAMA Netw Open* 3, e2013233. doi: 10.1001/jamanetworkopen.2020.13233

Vaidyanathan, U., Nelson, L. D., and Patrick, C. J. (2012). Clarifying domains of internalizing psychopathology using neurophysiology. *Psychol Med* 42, 447–459. doi: 10.1017/S0033291711001528

Wenzel, E. S., Eisenlohr-Moul, T., Nagelli, U., Bernabé, B. P., and Maki, P. M. (2022). Using self-report RDoC measures to identify transdiagnostic translational targets for perinatal affective disorders. *Arch Womens Ment Health* 25, 411–420. doi: 10.1007/s00737-021-01190-9

Woody, M. L., and Gibb, B. E. (2015). Integrating NIMH Research Domain Criteria (RDoC) into Depression Research. *Curr Opin Psychol* 4, 6–12. doi: 10.1016/j.copsyc.2015.01.004
